# Heme oxygenase 1 (HO-1) is a drug target for reversing cisplatin resistance in non-small cell lung cancer

**DOI:** 10.1016/j.jare.2025.05.033

**Published:** 2025-05-17

**Authors:** Jie Mei, Hui-Xiang Tian, Xiao-Ye Zhang, Yuan-Shen Chen, Lei-Yun Wang, Zhao Zhang, Yu-Long Zhang, Ding-Chao Rong, Jun Zeng, Min Dong, Yang Gao, Ji-Ye Yin, Hai-Jun Wu, Peng-Yuan Wang, Wei Zhang

**Affiliations:** a Department of Clinical Pharmacology, Xiangya Hospital, Institute of Clinical Pharmacology, Hunan Key Laboratory of Pharmacogenetics and National Clinical Research Center for Geriatric Disorders, Engineering Research Center of Applied Technology of Pharmacogenomics (Ministry of Education, China), Key Laboratory of Pharmacomicrobiomics of Hunan Province, Central South University, Changsha 410078, People's Republic of China; b Central Laboratory of Hunan Cancer Hospital, Central South University, Changsha 410013, People's Republic of China; c FuRong Laboratory, Changsha 410078 Hunan, People's Republic of China; d Oujiang Laboratory, Key Laboratory of Alzheimer’s Disease of Zhejiang Province, Institute of Aging, Wenzhou Medical University, Wenzhou 325000, People's Republic of China; e The First Affiliated Hospital of Guangdong Pharmaceutical University, Guangzhou 510080, People's Republic of China; f Department of Pharmacy, Traditional Chinese and Western Medicine Hospital of Wuhan, Tongji Medical College, Huazhong University of Science and Technology, Wuhan 430022, People's Republic of China; g Central South University Xiangya Medical School, Changsha 410013, People's Republic of China; h Department of Orthopedics, The First Affiliated Hospital of Shaoyang University, Shaoyang 422000, People's Republic of China; i Department of Thoracic Surgery, Xiangya Hospital, Central South University, Changsha 410008, People's Republic of China; j Pharmaceutical College, Guangxi Medical University, Nanning 530021, People's Republic of China; k Department of Oncology, Xiangya Hospital of Central South University, Changsha 410008, People's Republic of China

**Keywords:** NSCLC, HO-1, Drug resistance, Ferroptosis, Nrf2 pathway

## Abstract

**Introduction:**

Platinum-based drugs, the most widely used chemotherapeutic drugs in clinical oncology, have long faced the problem of drug resistance, which is urgently in need of resolution. Identifying biomarkers of drug resistance may help reduce platinum resistance and improve therapeutic efficacy.

**Objectives:**

This study aims to identify potential biomarkers associated with the development of cisplatin resistance in non-small cell lung cancer (NSCLC) and explore mechanisms to overcome chemoresistance.

**Methods:**

NSCLC cisplatin resistance cell lines were constructed, and transcriptome sequencing was performed. Results were validated using Gene Expression Omnibus (GEO) and The Cancer Genome Atlas (TCGA) databases. Molecular docking, proteomics sequencing, and *in vitro* and *in vivo* experiments were conducted to evaluate the role of Heme Oxygenase 1 (HO-1) in cisplatin resistance.

**Results:**

NSCLC cisplatin resistance cell lines, GEO and TCGA data identified HMOX1, downstream of Nrf2, as a key drug resistance gene induced by cisplatin. Activation of the Nrf2/HO-1 pathway was found to induce ferroptosis resistance, a critical mechanism of cisplatin resistance. Candidate compounds SB 202190 and Nordihydroguaiaretic acid (NDGA) effectively reactivated ferroptosis by inhibiting HO-1, thereby increasing cisplatin sensitivity.

**Conclusion:**

The Nrf2/HO-1 pathway is a significant contributor to cisplatin resistance in NSCLC. Targeting HO-1 with SB 202190 and NDGA presents a promising strategy to overcome resistance and improve chemotherapy outcomes.

## Highlights

• HMOX1 serves as a pivotal gene mediating cisplatin resistance.
• Activation of the Nrf2/HO-1 pathway promotes cisplatin resistance by inhibiting ferroptosis.
• SB 202190 and NDGA are two small-molecule compounds that can assist cisplatin in overcoming drug resistance.
• SB 202190 and NDGA reverse drug resistance by targeting HO-1 protein, disrupting Nrf2 mediated resistance mechanisms and restoring ferroptosis sensitivity.

## Graphical abstract

**Figure:**
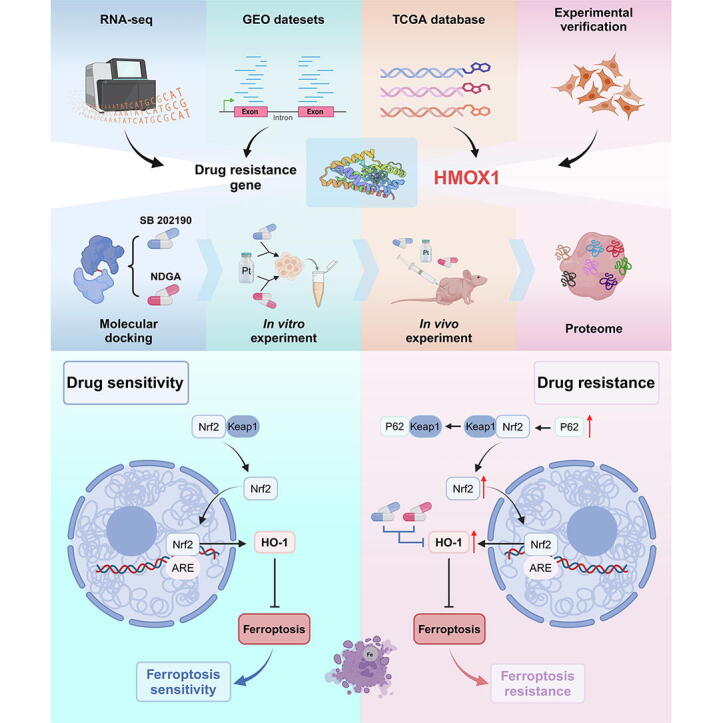

## Introduction

Lung cancer is the most commonly diagnosed cancer globally, accounting for approximately 12.4 % of all cancer cases, and deaths due lung cancer represent about one fifth of all cancer deaths, making it the leading cause of cancer mortality [1]. Of all lung cases, non-small cell lung cancer (NSCLC) is the most prevalent, representing 85 % [2]. The poor prognosis and low survival rate [3] of NSCLC make it a focal point in lung cancer research.

Currently, the treatment strategies for NSCLC mainly include chemotherapy, radiotherapy, immunotherapy, and surgery [3]. Although the continuous development of anti-tumor therapies has led to a plethora of emerging drugs such as targeted agents and immune checkpoint inhibitors [2], platinum drugs remain the first-line agents for NSCLC treatment [4,5]. In fact, clinically used immune drugs (PD-1 inhibitors, PD-L1 inhibitors, CTLA-4 inhibitors) [6], targeted drugs (e.g., EG-TKI inhibitors, ALK inhibitors) [7,8], etc., are often in combination with platinum drugs to achieve better anti-tumor effects. Notably, while platinum-based combination therapies have enhanced therapeutic efficacy, the pervasive challenge of platinum resistance continues to pose significant clinical obstacles, resulting in poorer prognoses for platinum-treated NSCLC patients [9]. Some studies have shown that the resistance mechanism may involve DNA damage repair [10], intracellular drug accumulation and metabolism [11,12], and oxidative stress [13], but the process of resistance development is still unclear.

Heme oxygenase 1 (HMOX1) encoded protein HO-1 is an oxygen-regulated protein that maintains cellular homeostasis by balancing the intracellular oxygen environment [14]. In contrast, the tumor tissue microenvironment typically exhibits a high reactive oxygen species (ROS) status [15], thus conferring a stronger adaptation to oxidative stress [16,17], which in turn to increased resistance of tumor cells to ROS-induced ferroptosis, autophagy, and other modes of cell death [18], and this is often the cause of drug resistance. Indeed, HO-1 has been reported to protect tumor cells from oxidative damage by scavenging ROS and reducing oxidative stress, thereby promoting their survival [19]. Furthermore, its role in rescuing cells from death is frequently implicated in the induction of drug resistance, with studies demonstrating that elevated HO-1 expression correlates with the development of chemoresistance [20]. Interestingly, HO-1 has also been reported to be a ferroptosis regulatory protein that can interfere with iron metabolism to regulate the process of ferroptosis [21].

Therefore, in this study, we performed transcriptome sequencing using autologous NSCLC cisplatin resistant cell lines, combined with drug resistant cell data from Gene Expression Omnibus (GEO) for validation, and used the The Cancer Genome Atlas (TCGA) data for clinical correlation analysis, ultimately screening out HMOX1 as a potential drug resistant gene. Both *in vitro* and *in vivo* experiments validated the critical role of HMOX1 in overcoming platinum resistance. Subsequently, small-molecule drugs were screened with HO-1 as the target, and SB 202190 and Nordihydroguaiaretic acid (NDGA) were to be potential targeted drugs. These candidate compounds can inhibit HO-1, thereby reversing cisplatin resistance through the Nrf2-mediated suppression of ferroptosis. This study not only proposes HMOX1 as a novel therapeutic target for platinum resistance but also elucidates its mechanistic basis and clinical relevance, providing the first evidence for this strategy in overcoming chemotherapy resistance.

## Materials and methods

### Cell culture

The human NSCLC cell lines A549, H23 and H460 were cultured in basic RPMI 1640 medium (Gbico, C11875500BT, Waltham, MA, USA), 10 % fetal bovine serum (Gbico, 10099141C, Waltham, MA, USA) and 1 % penicillin–streptomycin solution (Gbico, 15140122, Waltham, MA, USA). A549-R was induced by A549 cells and cultured in basic RPMI 1640 medium, 10 % fetal bovine serum, 1 % penicillin–streptomycin solution, and 2 μg/ml cisplatin (Sigma-Aldrich, P4394, St. Louis, MO, USA) to maintain its drug resistance.

### Construction of NSCLC cell lines with cisplatin resistance

The cisplatin resistance cell lines (A549-R, H23-R and H460-R) were developed by inducing A549, H23 and H460 cells with increasing concentrations of cisplatin, rendering them resistant. In short, 2 μg/ml cisplatin was added to A549, H23 and H460 cells for several days. Once the cells had adapted to growth, the cisplatin concentration in the complete medium was increased to 4 μg/ml. After appropriate time, the cisplatin-containing complete medium was changed to complete medium, and then a few days later, the cisplatin-containing complete medium was added to culture until the cells proliferate stably at this medium. Similarly, the concentration of cisplatin in the medium was increased to 6 μg/ml for culture and induction. The sensitivity of cells to cisplatin was measured during induction until the successful construction of cisplatin resistance cells, named A549-R, H23-R and H460-R.

### Cell proliferation assay

Cell proliferation was detected by cell counting kit-8 (CCK-8, Beyotime Biotechnology, C0038, Shanghai, China). The cells were seeded into 96-well plates at a density of 5000 cells per well. After 24 h of incubation, cells were treated with varying drug concentrations for specified durations. Then the medium was removed and 10 % CCK-8 solution was added, and the cells were incubated in a 37℃ incubator for 0.5–4 h. Optical density value at 450 nm was detected by Microplate Reader to calculate the survival rate of the cells.

### Overexpression of HMOX1

A549 cells were seeded into 6-well plates with 25w cells per well. Opti-MEM (Gibco, 31985–070, Waltham, MA, USA), lipofectamine 3000, p3000 (Thermo Fisher Scientific, L3000015, Waltham, MA, USA) and plasmids expressing HMOX1 (JTSBIO Co.,Ltd, Wuhan, China) were mixed and added into the 6-well plate, and the control vector was also added. The subsequent experiments were performed after transfection for appropriate time.

### Knockdown of HMOX1

A549-R cells were seeded into 6-well plates with 25w cells per well. Opti-MEM, Lipofectamine RNAiMAX (Thermo Fisher Scientific, 13778100, Waltham, MA, USA) and siRNA (named si596, si795 and si888, JTSBIO Co.,Ltd, Wuhan, China) of HMOX1 were mixed and added into the 6-well plate, the negative control (siNC) was also mixed and added. The subsequent experiments were performed after transfection for appropriate time.

### The combination of cisplatin, candidate compounds and various cell death inhibitors or ferroptosis inducers

A549-R and A549-R with HMOX1 konckdown cells were treated with cisplatin and NDGA (MedChemExpress, HY-N0198, Shanghai, China), SB 202190 (MedChemExpress, HY-10295, Shanghai, China) or (−)-Epicatechin (EC) (MedChemExpress, HY-N0001, Shanghai, China), to confirm whether the combination of these drugs can increase the lethality of cisplatin. In short, cisplatin, SB 202190, NDGA, and EC were separately added to A549-R and A549-R with HMOX1 konckdown cells. Meanwhile, cisplatin and NDGA/SB 202190/EC were co-administered to A549-R and A549-R with HMOX1 konckdown cells at different ratio. CCK-8 was used to detect the cell survival rate. The drug combination index (CI) was calculated by Chou-Talalay method [22]. Additionally, 20 μM apoptosis inhibitor (Z-VAD-FMK, S7023, Selleck, Houston, TX, USA), 20 μM necroptosis inhibitor (Necrostatin-1, S8037, Selleck, Houston, TX, USA), 1 mM autophagy inhibitor (3-Methyladenine, S2767, Selleck, Houston, TX, USA), 2.5 μM ferroptosis inhibitor (Ferrostatin-1, S7243, Selleck, Houston, TX, USA), 25 μM iron chelator (Deferoxamine mesylate, S5742, Selleck, Houston, TX, USA), 2 μM Erastin (S7242, Selleck, Houston, TX, USA) or 100 nM RSL3 (S8155, Selleck, Houston, TX, USA) and cisplatin/cisplatin + candidate compounds were co-treated 48 h, respectively.

### Reverse transcription quantitative PCR (RT-qPCR)

RNA extraction kit (GOONIE, 400–100, Guangzhou, China) was used to extract RNA from cells following the instructions suggested by the manufacturer. After RNA extraction, RNA concentration was detected and RNA quality was confirmed. The RNA was reverse transcribed into cDNA by reverse transcription kit (Takara Biomedical Technology (Beijing) Co., Ltd., RR047A, Beijing, China). Then cDNA, TB Green (Takara Biomedical Technology (Beijing) Co., Ltd., RR820A, Beijing, China) and primers were mixed for detection. The relative expression level was obtained by calculating 2^^-ΔΔCt^. The primers were listed in Table S1.

### Western blotting

The cell lysis buffer (RIPA Lysis Buffer: phosphatase inhibitor cocktail A: phenylmethanesulfonyl fluoride = 100:1:1) was added to the cells and lysed at 4 °C for 30 min, and the supernatant was centrifuged to obtain the protein. The protein concentration was quantified using BCA protein assay kit (Beyotime Biotechnology, P0010, Shanghai, China), and loading buffer was added to the protein and the mixture was denatured by heating. Then protein samples were separated by electrophoresis and transferred to PVDF membranes. After blocked, the membrane was incubated with primary antibodies overnight at 4 °C and horseradish peroxidase-conjugated secondary antibodies for 1 h at room temperature. Finally, the blots were captured after washing. The relative antibodies include: β-actin (Proteintech, 81115–1-RR, Rosemont, USA), HMOX1 (Proteintech, 10701–1-AP, Rosemont, USA), Nrf2 (Proteintech, 16396–1-AP, Rosemont, USA), GPX4 (Proteintech, 67763–1-Ig, Rosemont, USA), Keap1 (Proteintech, 10503–2-AP, Rosemont, USA) and P62 (Proteintech, 18420–1-AP, Rosemont, USA).

### Molecular docking

The X crystal diffraction structure (PDB ID: 6EHA) of HO-1 from RCSB PDB (https://www.rcsb.org/), which contains a large number of three-dimensional structures of proteins and nucleic acids. Meanwhile, the drug structures were obtained from the MCE Bioactive Compound Library Plus (containing 10.3 K compounds) databases. Schrodinger Maestro 11.4 software was used for molecular docking and PyMol was used for 3D mapping. The Virtual Screening Workflow module was used for virtual screening. The prepared compounds were imported, and the Glide module was used for molecular docking, and finally the compounds ranking was obtained.

### Animal experiments

Male nude mice of 4–5 week were raised in specific pathogen-free (SPF) environment for one week to adapt to the environment. A549 and A549-R cells were collected and counted. 100 μL cell suspension (5x10^7 cells/mL) was injected subcutaneously into the left upper limb armpit of nude mice to establish subcutaneous tumors. After the tumor volume reached 50–100 mm^3^, the mice injected with A459 cells were randomly divided into 2 groups: A549 control group and A549 + cisplatin group; The mice injected with A459-R cells were randomly divided into 5 groups: A549-R control group, A549-R + cisplatin group, A549-R + cisplatin + SB 202190 group, A549-R + cisplatin + NDGA group, and A549-R + cisplatin + EC group. Mice in the control group were intraperitoneally injected with normal saline. Mice in the cisplatin group was intraperitoneally injected with 3 mg/kg cisplatin. Mice in cisplatin + SB 202190 group were intraperitoneally injected 3 mg/kg cisplatin and 0.05 mg/kg SB 202190. Mice in cisplatin + NDGA group were intraperitoneally injected with 3 mg/kg cisplatin and 40 mg/kg NDGA simultaneously. And mice in cisplatin + EC group were intraperitoneally injected 3 mg/kg cisplatin and 5 mg/kg EC. The details are listed in Table S2. The drugs were administered once every 3 days for 8 times. The body weight of mice, the length and width of tumor were recorded every 3 days during the experiment. Theses mice were sacrificed after the tumor volume reached 800–1200 mm^3^. Tumors and other tissues were collected and measured. All animal experimental protocols were reviewed and approved by the Institutional Animal Care and Use Committee, Wenzhou Institute, University of Chinese Academy of Sciences (No. WIUCAS23011701).

### Histopathological analysis

The collected tissues were immersed in formalin for 24 h and then embedded in paraffin. The paraffin-embedded tissues were cut into slices of 3–5 μm thickness. For immunohistochemistry (IHC), sections were deparaffinized through xylene gradients, rehydrated via ethanol series, and subjected to antigen retrieval in EDTA buffer using microwave heating. After blocking endogenous peroxidase with 3 % hydrogen peroxide solution and nonspecific sites with 5 % goat serum, sections were incubated overnight at 4 °C with primary antibodies (TTF-1, Santa Cruz Biotechnology, sc-53136; Naspin A, Santa Cruz Biotechnology, sc-517223, Dallas, USA; P63, Servicebio, GB11396-1, Wuhan, China; HO-1, Proteintech, 10701–1-AP, Rosemont, USA) and corresponding secondary antibodies. After washing, the slices were stained with DAB and observed under a microscope. For hematoxylin-eosin (H&E) staining, the tissue slices were stained with hematoxylin and eosin.

### Detection of biochemical indexes in serum

The blood of the mice in each group was obtained before sacrifice. After placing at room temperature for 3 h, serum was isolated by centrifugation (2500 × g, 15 min, 4 °C) and stored at − 80 °C until analysis. The levels of alanine aminotransferase (ALT), aspartate aminotransferase (AST), blood urea nitrogen (BUN), creatinine (CREA), creatine kinase (CK), creatine kinase myocardial band (CKMB) were detected by automatic biochemical analyzer (Alanine aminotransferase assay kit, BIOBASE, 70211; aspartate aminotransferase assay kit, BIOBASE, 70210; blood urea nitrogen assay kit, BIOBASE, 70224; creatinine assay kit, BIOBASE, 70227; creatine kinase assay kit, BIOBASE, 70222; creatine kinase myocardial band assay kit, BIOBASE, 70223, Jinan, China).

### Transmission electron microscope (TEM)

A549 cells were treated with cisplatin (8 μM). A549-R cells were treated with cisplatin (8 μM), cisplatin (8 μM) combined with SB 202190 (8 μM), cisplatin (8 μM) combined with NDGA (16 μM), and cisplatin (8 μM) combined with EC (48 μM) for 48 h. Cells were collected and fixed in 2.5 % glutaraldehyde overnight at 4℃. After washing with PBS, 1 % osmium tetroxide was added and fixed again. The cells were placed in gradient ethanol, acetone, the mixture of acetone and resin, and embedded in resin, which was cut into 70–90 nm slices after gradient heating. The slices were stained with uranyl acetate and lead citrate, and dried for microscope observation.

### Inductively coupled plasma-mass spectrometry (ICP-MS) detection

A549 cells were treated with cisplatin (10 μM). A549-R cells were treated with cisplatin (10 μM), cisplatin (10 μM) combined with SB 202190 (20 μM), cisplatin (10 μM) combined with NDGA (20 μM), and cisplatin (10 μM) combined with EC (120 μM) for 48 h. Cells were collected and added with HNO_3_. Then the samples were digested in graphite digester, and added with H_2_O_2_ to continue digestion. After cooling, the sample was diluted with ultrapure water to 10 ml for detection.

### Transcriptome analysis

Total RNA was extracted with TRIzol reagent (Invitrogen, CA, USA, Total RNA Extraction Kit 2.0 Plus, R411-C3) from cell samples of different groups. The purity and quantification of RNA were determined using a NanoDrop 2000 spectrophotometer (Thermo Scientific, USA), and RNA integrity was assessed with an Agilent 2100 Bioanalyzer (Agilent Technologies, Santa Clara, CA, USA). Transcriptome libraries were constructed following the manufacturer’s protocol using the VAHTS Universal V6 RNA-seq Library Prep Kit. Sequencing was performed on an Illumina NovaSeq 6000 platform. Raw reads in FASTQ format were processed using fastp (version 0.20.1) to remove low-quality reads, resulting in clean reads. The clean reads were mapped to the reference genome using HISAT2 (version 2.1.0). Gene expression levels were quantified as FPKM, and read counts for each gene were obtained using HTSeq-count (version 0.11.2). Differentially expressed genes (DEGs) were identified using DESeq2 (version 1.22.2) with a fold change threshold of > 4 or < 0.25 and P-value < 0.005. Radar plots were generated using the R package ggradar. Functional enrichment analyses, including Gene Ontology (GO), Kyoto Encyclopedia of Genes and Genomes (KEGG), Reactome and WikiPathways, were performed to identify significantly enriched functional terms. Bar plots, and enrichment circle plots of significantly enriched terms were visualized using R (v4.0.3). Gene Set Enrichment Analysis (GSEA) was performed using the GSEA software (version 4.3.3, Broad Institute, USA) to identify significantly enriched pathways associated with DEGs.

### LC-MS/MS analysis

The mice tumors (100 mg) were rapidly ground in liquid nitrogen and lysed with lysis buffer for total protein extraction. After the quantification of BCA kit, a part of the protein was taken for sodium dodecyl sulfate–polyacrylamide gel electrophoresis. Another part of protein was enzymolized by trypsin, and finally phosphoric acid was added to regulate PH to terminate the reaction. The samples were desalted on SOLA™ SPE and dried under vacuum. Before detection, the samples were resuspended and added with iRT peptides (1:20), and then detected by LC-MS/MS. All analyses were performed by a Tims TOF Pro mass spectrometer (Thermo, Bruker) equipped with an Easyspray source (Thermo, USA). DIA technology was used to collect the mass spectrum data of each sample for spectral matching, quantitative information extraction and statistical analysis. Raw data was processed using Spectronaut Pulsar 18.7 software (Biognosys, Swiss). The search sequence file is uniprot-Homo sapiens-9606–2024.2.1.fasta (https://www.uniprot.org/). After protein annotation and quantification, the thresholds of fold change (>2 or < 0.5) and P value < 0.05 were used to identify differentially expressed proteins (DEPs). DEPs were further used for GO and KEGG enrichment analysis. Protein-protein interaction analysis was performed using the STRING (version: 12.0, https://cn.string-db.org/). The key positive and negative regulatory pathways were analyzed by Gene set enrichment analysis (GSEA) software.

### GEO data analysis

Datasets containing transcriptomic data of NSCLC cell line and cisplatin resistance NSCLC cell line were retrieved from the GEO database. GSE108214 and GSE84146 were selected for further analysis. The GSE108214 dataset includes transcriptomic data from A549 cisplatin sensitivity (A549-s) and A549 cisplatin resistance (A549-r) cell lines. The GSE84146 dataset comprises transcriptomic data from H23 cisplatin sensitivity (H23-s), H23 cisplatin resistance (H23-r), H460 cisplatin sensitivity (H460-s) and H460 cisplatin resistance (H460-r). The platform files for these datasets are GPL17077 (Agilent-039494 SurePrint G3 Human GE v2 8x60K Microarray 039381) and GPL6480 (Agilent-014850 Whole Human Genome Microarray 4x44K G4112F), respectively. Data processing and visualization analyses were conducted using R (v4.0.3).

### TCGA data analysis

We downloaded STAR-counts data and corresponding clinical information for 1017 tumors from the TCGA database (https://portal.gdc.cancer.gov). We then extracted data in TPM format and performed normalization using the log_2_(TPM + 1) transformation. We use the log rank test to compare the survival differences between the two groups mentioned above in the KM survival analysis. For the Kaplan-Meier curve, the p-value and hazard ratio with a 95 % confidence interval are obtained through the logrank test and univariate Cox regression. Visualization of the results was performed using the R packages ggrisk, survival and survminer. All analytical methods above and R packages were performed using R (v4.0.3). P < 0.05 was considered as statistically significant.

### Statistical analysis

All data analyses were conducted using SPSS version 24.0 (SPSS Inc., Chicago, IL, USA). The data are expressed as mean ± standard deviation (SD). For comparisons between two groups, Student’s *t*-test was applied. One-way ANOVA or two-way ANOVA was utilized for multi-group analyses. Statistical significance was defined as P < 0.05.

## Results

### HMOX1 is overexpressed in cisplatin resistance cell line

**Fig. 1:**
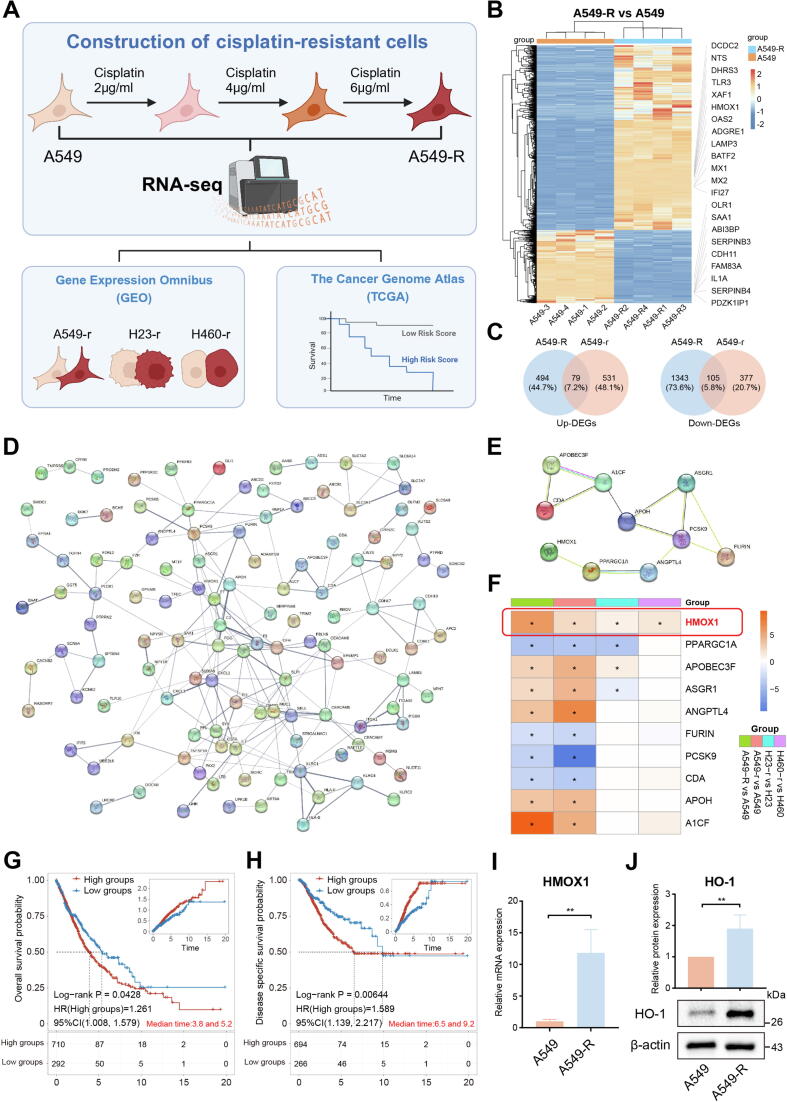
Construction of cisplatin resistance cell line A549-R and transcriptome analysis. (A) Transcriptome sequencing was conducted on A549 and A549-R cells, followed by integration and analysis of data from GEO and TCGA databases. (B) The heatmap of DEGs (A549-R vs A549). (C) The shared DEGs from our transcriptome data and GSE108214. (D) The PPI network of shared DEGs. (E) The top 10 hub genes were screening from PPI network. (F) Among the hub genes, only HMOX1 was consistently upregulated in cisplatin resistance cell lines in our transcriptome sequencing, GSE108214 and GSE84146. Low HMOX1 expression is associated with better (G) overall survival and (H) disease-specific survival. (I) RT-qPCR and (J) western blot showed HMOX1 was upregulated in A549-R. All experiments were independently repeated at least three times.

Firstly, the A549 cell line was progressively induced into the cisplatin resistance A549-R cell line by stepwise stimulation with increasing low concentrations of cisplatin. The IC50 value of A549 and A549-R were: 8.53 ± 0.15 μM and 22.10 ± 1.30 μM, respectively (Fig. S1A). To identify key genes involved in cisplatin resistance, transcriptome sequencing was performed on A549 and A549-R cell lines, combined with integrative analysis of GEO and TCGA data (Fig. 1A). Principal component analysis (PCA) and correlation box plots were shown in Fig. S1B and Fig. S1C. A total of 2021 DEGs were identified (Fig. 1B). Pathway enrichment analysis was performed on these DEGs (Fig. S1D, E). To identify core genes regulating cisplatin sensitivity, a protein–protein interaction (PPI) network was constructed based on the 2021 DEGs, and the core submodule were identified, screening the top 10 hub genes, including HMOX1, TNF, ACE, ATP4A, ATP12A, MAOA, SNCA, GRIN2B, NCAM1 and PRKN (Fig. S2A-C).

Further validation of core genes implicated in cisplatin resistance was pursued through a systematic search of the GEO database, focusing on cisplatin sensitivity and resistance datasets. Two datasets, GSE108214 and GSE84146, were selected for subsequent association analysis. GSE108214 contains RNA-sequencing data from NSCLC cell lines sensitive to cisplatin (A549-s) and resistant to cisplatin (A549-r) (Fig. S3A), with 1092 DEGs identified (Fig. S3B). GO and KEGG enrichment analysis of these DEGs was shown in Fig. S3C. A total of 184 identical DEGs were shared between our transcriptomic data and GSE108214 (Fig. 1C), and the corresponding PPI network was shown in Fig. 1D. The top 10 hub genes were identified, including HMOX1, PPARGC1A, ANGPTL4, PCSK9, FURIN, ASGR1, APOH, A1CF, CDA and APOBEC3F (Fig. 1E). Analysis of the GSE84146 dataset, comprising cisplatin sensitive (H23-s, H460-s) and cisplatin resistance (H23-r, H460-r) NSCLC cell lines, revealed consistent upregulation of HMOX1 across cisplatin resistance cell lines (Fig. 1F).

Drug resistance is a significant factor in poor prognosis. The consistent upregulation of HMOX1 in cisplatin resistance cell lines suggests that HMOX1 may be a risk gene for poor prognosis. TCGA data were selected to further investigate the role of HMOX1 in the cisplatin resistance within NSCLC patients. The results showed that HMOX1 were correlated with the prognosis of NSCLC patients (Fig. S4). Patients with low HMOX1 expression had better overall survival (Fig. 1G) and disease-specific survival (Fig. 1H), suggesting that HMOX1 may act as a drug resistance gene affecting patient outcomes. Finally, the expression of HMOX1 was validated in the self-established cisplatin resistance cell line A549-R, confirming that both the RNA and protein expression levels of HMOX1 increased in A549-R (Fig. 1I, J). Furthermore, the expression of HMOX1 in other NSCLC cell lines with cisplatin resistance (H23-R and H460-R) were validated, which revealed consistent findings that both H23-R and H460-R exhibited significantly upregulated HMOX1 expression (Fig. S5). Additionally, other hub genes did not exhibit consistent expression in A549-R, H23-R and H460-R cell lines (Fig. S6). All results confirmed that HMOX1 was highly expressed in cisplatin resistance cells and was associated with poor prognosis of NSCLC patients, suggesting that HMOX1 may serve as a potential therapeutic target to improve patient treatment outcomes.

### HMOX1 as a drug resistance gene

**Fig. 2:**
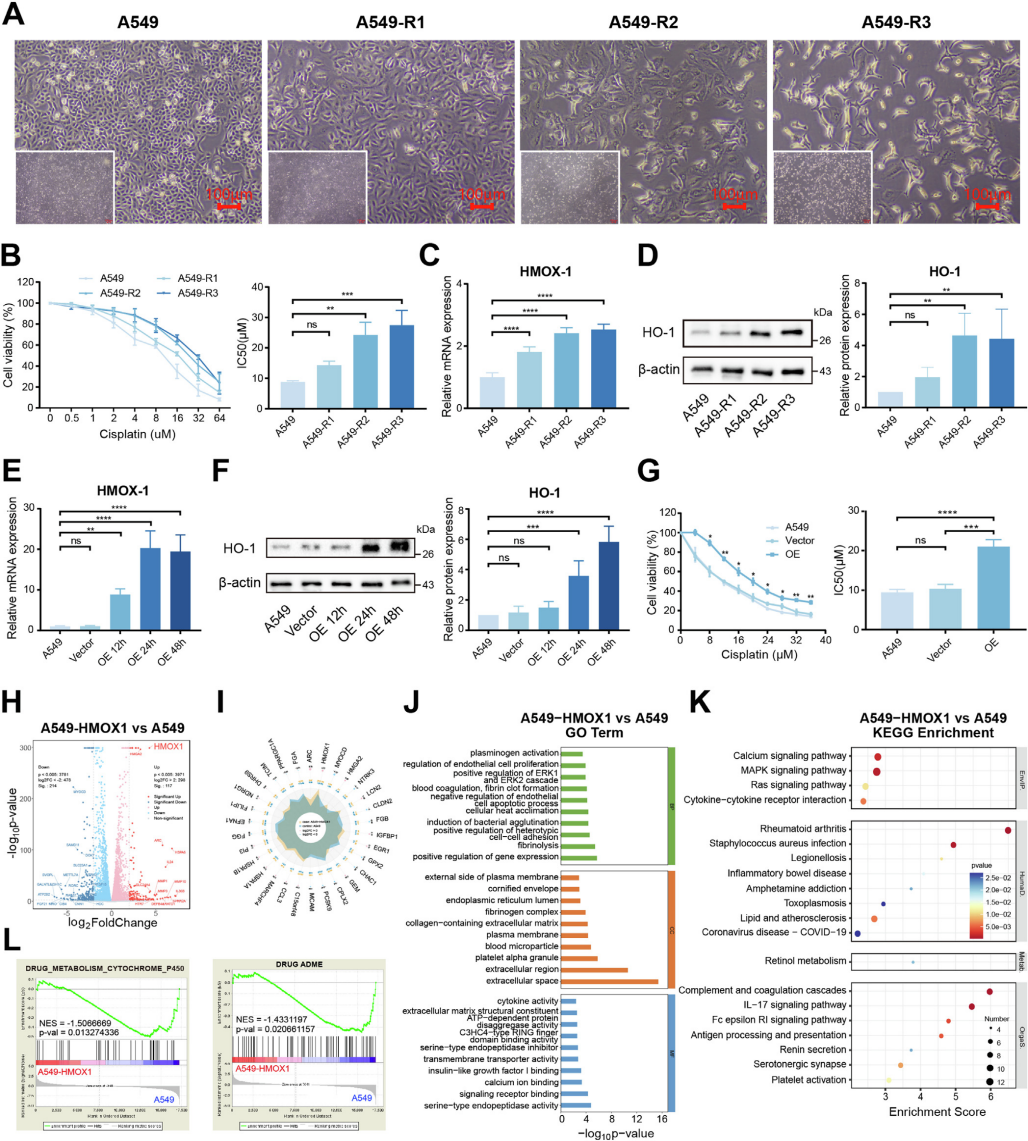
HMOX1 overexpression and transcriptome analysis. (A) Changes in cell morphology during the induction of A549-R. (B) Drug resistance gradually developed during the induction of A549-R. (C) RT-qPCR and (D) western blotting showed that the expression of HMOX1 gradually increases during the induction of A549-R. (E) RT-qPCR and (F) western blotting demonstrated successful HMOX1 overexpression in A549. (G) Overexpression of HMOX1 led to cisplatin resistance in A549. (H) Volcano plot, (I) radar plot, (J) GO, (K) KEGG enrichment and (L) GSEA analysis of DEGs (A549-HMOX1 vs A549). All experiments were independently repeated at least three times.

Interestingly, distinct morphological alterations emerged in A549-R cells throughout the induction process (Fig. 2A). As the duration and dosage of cisplatin stimulation increased, drug resistance gradually escalated (Fig. 2B), which paralleled the progressive upregulation of HMOX1 expression at both transcriptional and translational levels (Fig. 2C, D). In addition, plasmids were transfected into A549 cells to induce HMOX1 overexpression (Fig. 2E, F). Strikingly, forced HMOX1 expression significantly enhanced cisplatin resistance (Fig. 2G), providing direct experimental evidence that HMOX1 acts as a cisplatin resistance gene.

To investigate the role of HMOX1 in drug resistance mechanisms, transcriptomic analysis was conducted between A549 cells and A549-HMOX1 (HMOX1-overexpressing in A549 cells). PCA and correlation box plots, as depicted in Fig. S7A and B, demonstrated distinct transcriptional profiles between the two cell lines. Differential expression analysis revealed 331 significantly altered genes following HMOX1 overexpression (Fig. 2H), with radar plot highlighting HMOX1 as the most markedly upregulated gene (Fig. 2I). Enrichment analyses (Fig. 2J, K) and PPI network (Fig. S8A-C) of these DEGs are performed. HMOX1, PPARGC1A, and TNF were identified as hub genes, consistent with previous analysis. GSEA analysis revealed that HMOX1 overexpression inhibited drug metabolism and ADME (Fig. 2L). Interestingly, while HMOX1 overexpression increased drug resistance, it also activated pathways associated with autophagy, apoptosis and necroptosis (Fig. S9A). Meanwhile, HMOX1 overexpression altered cellular oxygen response pathways, suppressed lipid peroxidation pathways (Fig. S9B, C), altered iron ion homeostasis (Fig. S9D) and inhibited ferroptosis pathways (Fig. S9E), providing insights into the mechanism by which HMOX1 contributes to cisplatin resistance.

### Inhibition of HO-1 alleviates cisplatin resistance

**Fig. 3:**
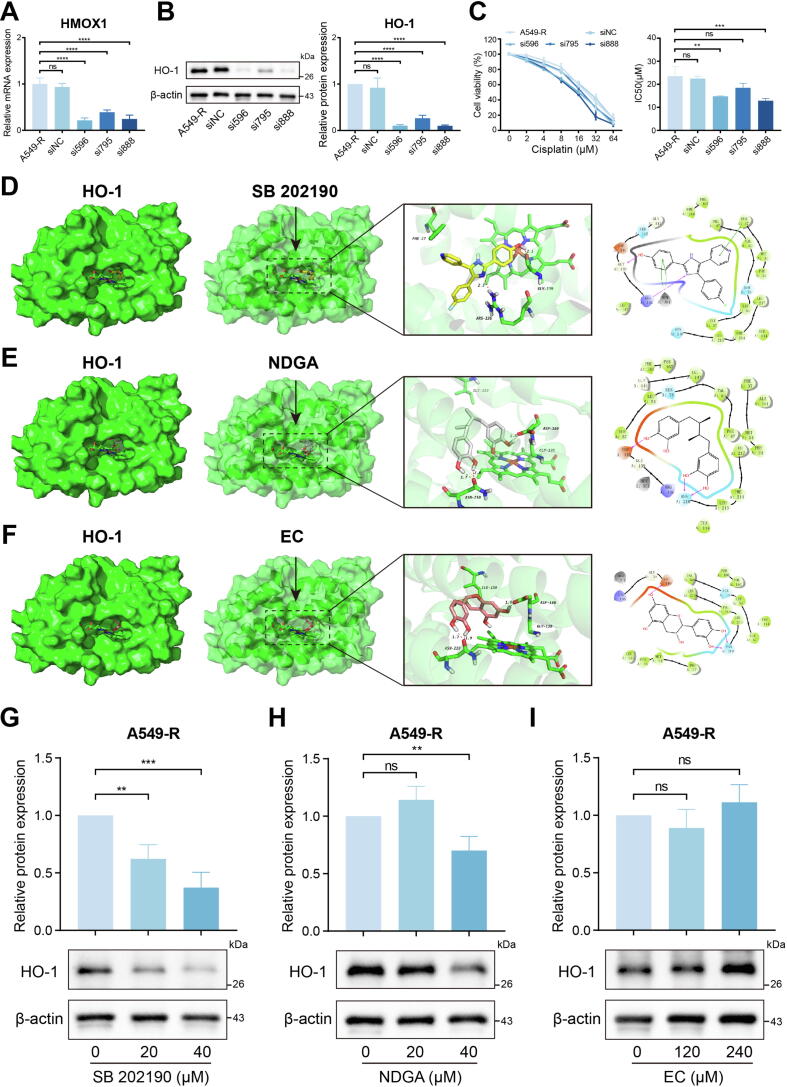
Molecular docking and validation based on HO-1. (A) RT-qPCR and (B) western blotting verified the knockdown efficiency of three HMOX1 siRNA in A549-R. (C) After HMOX1 knockdown, CCK-8 assays were performed to assess the survival rate of A549-R to cisplatin. Detailed docking information of (D) SB 202190, (E) NDGA and (F) EC with HO-1. Western blotting verified the regulation of HO-1 by (G) SB 202190, (H) NDGA and (I) EC. All experiments were independently repeated at least three times.

While HMOX1 has been implicated in cisplatin resistance, its therapeutic potential for resistance reversal remains unexplored. To investigate this, three distinct HMOX1-targeting siRNAs (si596, si795, and si888) were designed and validated, all demonstrating efficient knockdown of HMOX1 expression in A549-R cells (Fig. 3A, B). Among them, si596 and si888 exhibited the highest knockdown efficiency, achieving over 80 % knockdown within 48 h (Fig. S10A, B). Knocking down HMOX1 increased the sensitivity of A549-R cells to cisplatin (Fig. 3C), suggesting that regulation of HMOX1 expression can alleviate cisplatin resistance.

Subsequently, a comprehensive virtual screening analysis was conducted to identify potential small-molecule modulators of HO-1 (encoded by HMOX1). The structure of HO-1 was retrieved from the Protein Data Bank (Fig. S11A), and molecular docking was performed against a library of 10,300 compounds. The top 100 compounds, based on docking scores, were systematically evaluated (Table S3). Among these, three compounds (SB 202190, NDGA and EC) emerged as promising candidates (Fig. S11B-D), all of which have been reported to possess potential for regulating HO-1 in various studies [[23], [24], [25]]. SB 202190 was found to form two hydrogen bonds, three π-π interactions, and several hydrophobic interactions with HO-1 (Fig. 3D). NDGA demonstrated the ability to form three hydrogen bonds and multiple hydrophobic interactions with HO-1 (Fig. 3F). EC formed three hydrogen bonds and several hydrophobic interactions with HO-1 (Fig. 3G). Finally, experiments verified that SB 202190 significantly inhibited HO-1 expression (Fig. 3H). NDGA showed inhibitory effects on HO-1 at higher doses (Fig. 3I), while EC did not exhibit significant inhibition of HO-1 expression in this study (Fig. 3J).

### The combination of candidate compounds and cisplatin can overcome cisplatin resistance

**Fig. 4:**
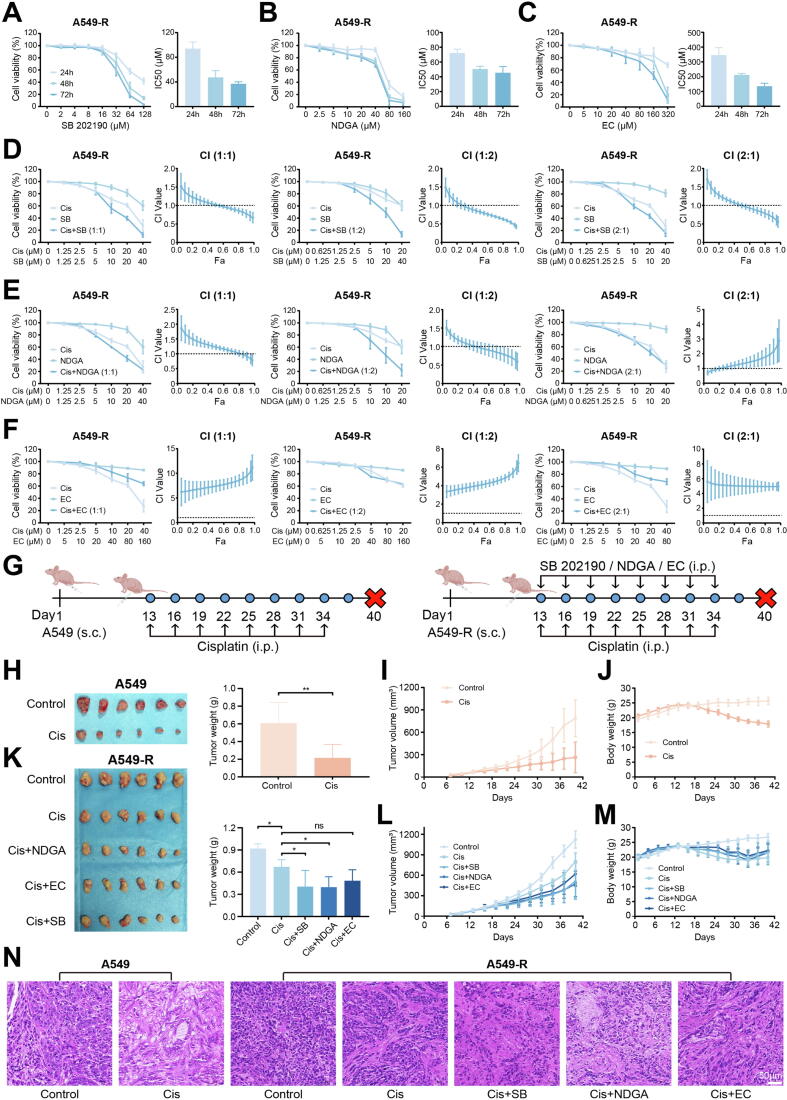
The *in vivo* and *in vitro* combined effects of candidate drugs with cisplatin. The survival rates and IC50 of A549-R when treated with (A) SB 202190, (B) NDGA and (C) EC individually. The effects of different ratios of (D) SB 202190, (E) NDGA or (F) EC in combination with cisplatin. All experiments were independently repeated at least three times. (G) Schematic illustration of experiments in A549 and A549-R xenograft tumor models. (H) Tumor images and tumor weight of each group (n = 6) in A549 xenograft tumor models. (I) Tumor volume and (J) body weight of each group during administration in A549 xenograft tumor models. (K) Tumor images and tumor weight of each group (n = 6) in A549-R xenograft tumor models. (L) Tumor volume and (M) body weight of each group during administration in A549 xenograft tumor models. (N) H&E staining of tumor tissues of each group.

Firstly, the three candidate compounds (SB 202190, NDGA, and EC) each exhibited potent anti-proliferative activity in A549-R cells (Fig. 4A-C). The IC50 values of 48 h for SB 202190, NDGA, and EC were 47.46 μM, 50.50 μM, and 211.93 μM, respectively (Fig. 4A-C). Subsequent evaluation of fixed-ratio combinations between candidate compounds and cisplatin was performed in A549-R cells, with CI values calculated to quantify drug combinations (CI = 1: additive effect; CI < 1: synergy; CI > 1: antagonism). The results revealed that both SB 202190 (Fig. 4D) and NDGA (Fig. 4E) exhibited synergistic effects with cisplatin, whereas EC showed antagonism with cisplatin. (Fig. 4F).

*In vivo* experiments, A549 and A549-R xenograft tumor models in nude mice, were also conducted to assess the potential of candidate compounds. The schedule of experiments was shown in Fig. 4G. Cisplatin (3 mg/kg) alone or in combination with SB 202190 (0.05 mg/kg), NDGA (40 mg/kg) or EC (5 mg/kg) were administered to mice every three days. In A549 xenograft models, cisplatin demonstrated potent antitumor activity (Fig. 4H), achieving a 64.6 % tumor inhibition rate (Fig. 4I). However, a significant weight loss in mice was observed following cisplatin administration (Fig. 4J), suggesting that cisplatin caused notable side effects. The therapeutic efficacy of cisplatin was markedly attenuated in A549-R xenograft models (Fig. 4K), showing only a 26.8 % tumor inhibition rate (Fig. 4L), confirming the development of cisplatin resistance. Importantly, combination therapy with either SB 202190 or NDGA significantly restored cisplatin sensitivity in resistant tumors, whereas EC failed to enhance cisplatin's antitumor effects (Fig. 4K, L). Weight loss was also observed in the mice treated with cisplatin, and the addition of candidate compounds did not significantly ameliorate the weight loss (Fig. 4M). IHC tumor tissues showed positive staining of Napsin A and TTF1, and the absence of P63 expression, which are characteristic pathological features of lung adenocarcinoma (Fig. S12), indicating successful mice models establishment. Consistent with previous findings, the expression of HO-1 was higher in tumor tissues from the A549-R mice model group compared to the A549 mice model group (Fig. S13). H&E staining of tumor tissues showed active growth and tight cell arrangement in both A549 and A549-R mice models, with vacuolization occurring after cisplatin treatment. The addition of candidate compounds enhanced this vacuolar phenotype (Fig. 4N). Statistical analysis of organ weights in the heart, liver, spleen, lung and kidney tissues of mice revealed that the candidate compounds did not significantly alter the organ indices of the mice (Fig. S14). H&E staining of spleens showed that candidate compounds improved the spleen toxicity induced by cisplatin (Fig. S15). However, pathological analysis of the heart, liver, lung and kidney tissues of mice did not reveal any significant impact of the candidate compounds on the histopathology of these organs (Fig. S16). Biochemical analysis in serum showed that candidate compounds did not affect the hepatic function (ALT, AST) and cardiac function (CK, CK-MB) (Fig. S17A, B). Moreover, cisplatin administration significantly increased BUN levels (renal function), and both SB 202190 and NDGA partially alleviated cisplatin-induced renal toxicity (Fig. S17C). These findings preliminarily confirm that SB 202190 and NDGA have the potential to enhance the efficacy of cisplatin and overcome drug resistance without inducing additional toxic side effects.

### Ferroptosis inhibition as a major mechanism of cisplatin resistance

**Fig. 5:**
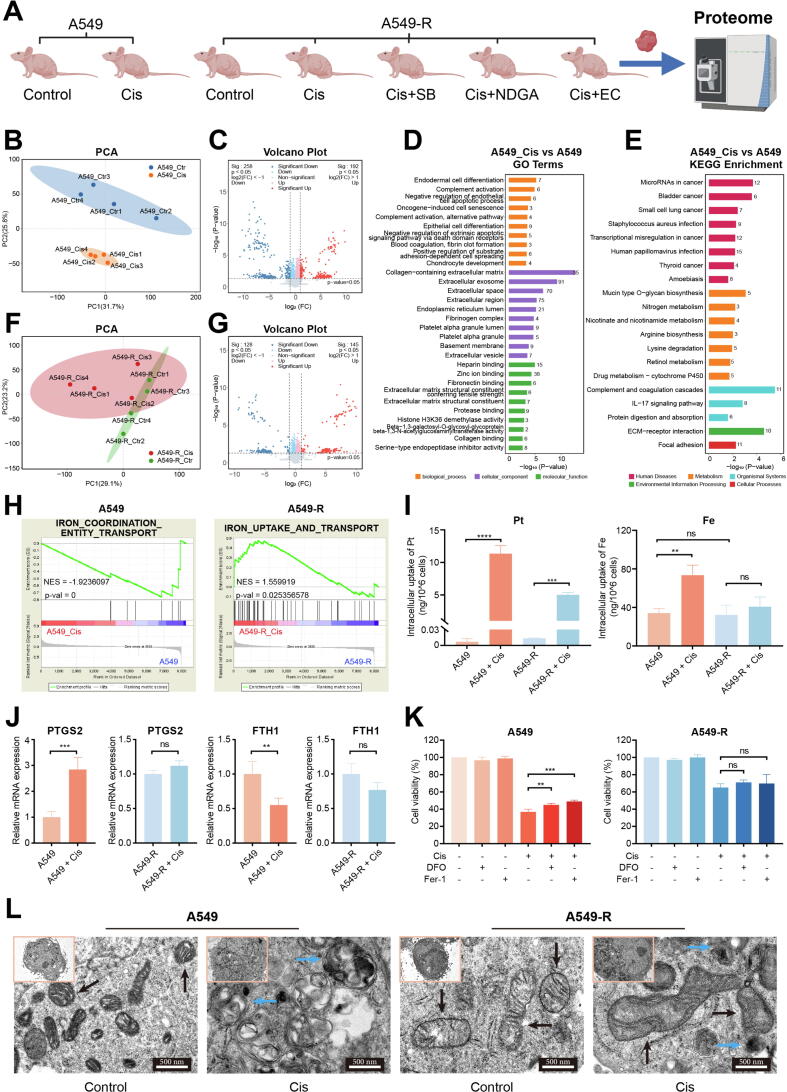
Ferroptosis inhibition led to cisplatin resistance. (A) Schematic diagram of the tumor samples for proteomic sequencing. (B) PCA and (C) volcano plot of proteomic data in A549 + cisplatin and A549 group. (D) GO and (E) KEGG enrichment analysis of DEGs in A549 + Cisplatin and A549 group. (F) PCA and (G) volcano plot of proteomic data in A549-R + cisplatin and A549-R group. (H) GSEA exhibited that cisplatin administration was related to iron transport. (I) Platinum and iron content were measured in different group. (J) Ferroptosis biomarkers (PTGS2 and FTH1) were detected in different group. (K) Ferroptosis inhibitors (Ferrostatin-1, Fer-1) and iron chelator (Deferoxamine, DFO) reversed the cisplatin-induced cell death in A549, but not in A549-R. (L) Representative TEM images of different group. The black arrows indicate normal mitochondria, the red arrows indicate the abnormal mitochondria, and the blue arrows indicate the apoptotic body. All experiments were independently repeated at least three times. (For interpretation of the references to colour in this figure legend, the reader is referred to the web version of this article.)

To elucidate the mechanistic role of HMOX1 and candidate compounds in cisplatin resistance, proteomic sequencing was conducted on tumor tissues from the animal experiments (Fig. 5A). The plot of sample expression abundance and tree plot are shown in Fig. S18A, B. Cisplatin treatment significantly impacted protein expression in A549 xenograft tumor models (Fig. 5B), resulting in 450 DEPs (Fig. 5C). The primary effects of cisplatin were concentrated in DNA damage and apoptosis (Fig. 5D). Additionally, pathways related to drug metabolism-cytochrome P450 and extracellular matrix (ECM) were also notably enriched (Fig. 5E). In contrast, in A549-R xenograft tumor models, the effects of cisplatin were significantly suppressed (Fig. 5F), resulting in 273 DEPs (Fig. 5G). Enriched pathways are presented in Fig. S19A, B. Interestingly, only 47 proteins (14.2 %) showing consistent regulation after cisplatin administration in A549 and A549-R xenograft tumor models, indicating that effects of cisplatin were markedly different in the two groups (Fig. S20A).

GSEA demonstrated consistent activation of traditional apoptotic pathways by cisplatin treatment in both A549 and A549-R xenograft tumors (Fig. S20B). Furthermore, although A549-R showed cisplatin resistance, cisplatin still activated autophagy and necrosis-related signaling pathways (Fig. S20C), suggesting that A549-R resist cisplatin-induced cell death through alternative mechanisms. Considering the role of HO-1 in redox balance and iron homeostasis, further investigation was focused on ferroptosis-associated pathways. In A549 xenograft tumor models, cisplatin significantly activated ROS biosynthesis pathways (Fig. S20D), while in A549-R xenograft tumor models, cisplatin induced more activation in oxygen transport and oxygen binding pathways (Fig. S20E), as well as ROS metabolism pathways (Fig. S20F). Regarding iron transport, after cisplatin administration, A549-R xenograft tumor models exhibited a stronger enrichment in iron transport pathways compared to A549 xenograft tumor models (Fig. 5H). Interestingly, cisplatin activated the Nrf2 pathway in A549, but inhibited the pathway in A549-R, despite not reaching statistical significance (Fig. S20G).

Analysis of metal ion content in cisplatin-treated A549 and A549-R cell lines revealed significantly elevated intracellular platinum levels in both cell types (Fig. 5I), which is the main cause of apoptosis. Notably, cisplatin significantly increased intracellular iron levels within A549, while this effect was markedly inhibited in A549-R (Fig. 5I). Further examination of ferroptosis biomarkers demonstrated that cisplatin treatment in A549 cells significantly upregulated PTGS2 expression while downregulating FTH1 expression. In contrast, these effects were not observed in A549-R cells (Fig. 5J). Subsequent experiments confirmed that the ferroptosis inducers (Erastin and RSL3) did not affect cisplatin-induced cell death in A549 (Fig. S21), whereas ferroptosis inhibitors (Ferrostatin-1, Fer-1) and the iron chelator (Deferoxamine mesylate, DFO) effectively rescued cisplatin-induced cell death in A549 cells but showed no rescuing effect in A549-R cells (Fig. 5K). These results indicated that cisplatin induces ferroptosis in the A549, while this effect was suppressed in the A549-R. Excessive iron within cells leads to ferroptosis, primarily characterized by mitochondrial damage and dysfunction. TEM results showed that, compared to A549, the cisplatin treatment group exhibited irregular mitochondrial morphology, increased vacuolization, mitochondrial membrane collapse, and a reduction in mitochondrial cristae (Fig. 5L). In contrast, A549-R + cisplatin group showed relatively normal mitochondria, with intact morphology and clear cristae, without significant mitochondrial dysfunction, suggesting that cisplatin treatment mitigated mitochondrial damage in A549-R (Fig. 5L). These results suggest that in A549-R, cisplatin-induced ferroptosis was suppressed. And iron homeostasis dysregulation and the inhibition of ferroptosis contributed to the development of cisplatin resistance.

### Role of Nrf2/HO-1 pathway in ferroptosis inhibition

**Fig. 6:**
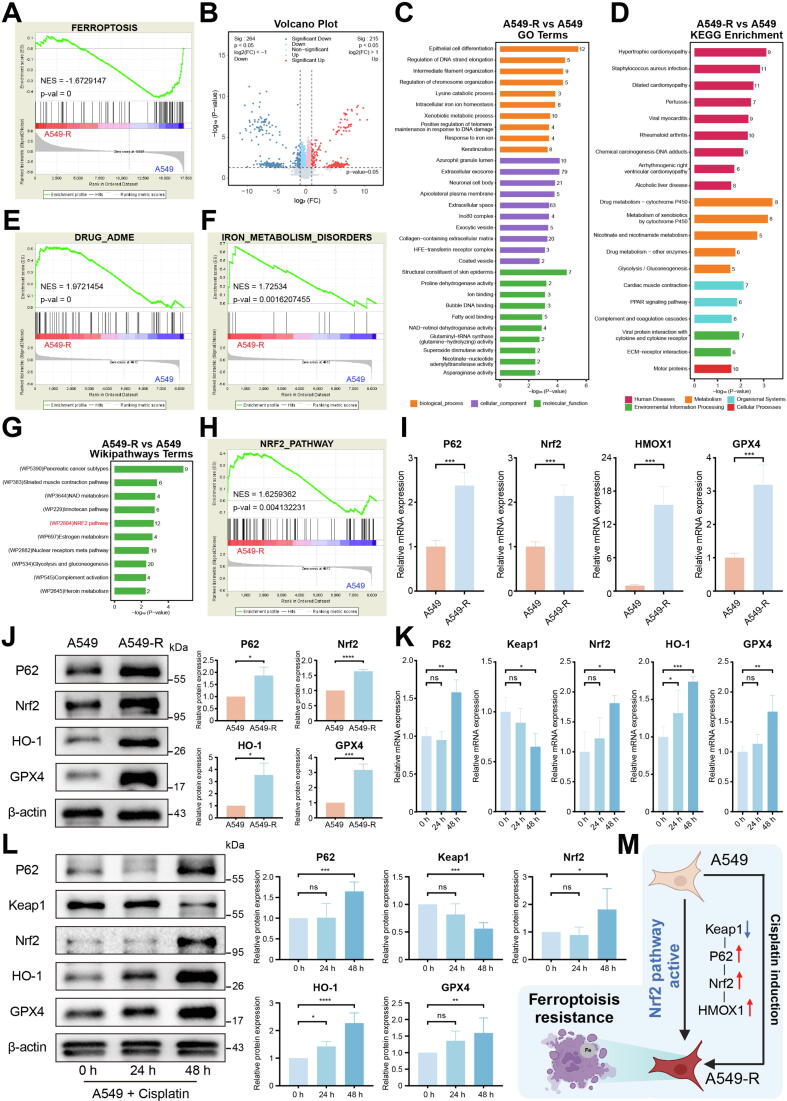
The activation of the Nrf2 pathway mediates ferroptosis resistance. (A) GSEA showed that the ferroptosis pathway was inhibited in A549-R. (B) Volcano plot of DEPs in A549-R and A549 xenograft tumor models. (C) GO and (D) KEGG enrichment analysis of these DEPs. GSEA showed that DEPs were enriched in (E) drug ADME and (F) iron metabolism disorders. (G) Wikipathways and (H) GSEA showed that there DEPs were enriched in Nrf2 pathway. (I) RT-qPCR and (J) western blotting showed that P62, Nrf2, HO-1 and GPX4 were upregulated in A549-R. (K) RT-qPCR and (L) western blotting showed that P62, Nrf2, HO-1 and GPX4 were upregulated, and Keap1 was down-regulated in A549-R. (M) The mechanism diagram of Nrf2/HMOX1-mediated cisplatin resistance. All experiments were independently repeated at least three times.

By performing an integrative analysis of the transcriptomic and proteomic data from A549 and A549-R, the mechanisms underlying iron homeostasis and ferroptosis in the development of cisplatin resistance were explored. Transcriptomic analysis of A549-R and A549 cells revealed that A549-R was enriched in apoptosis and necroptosis (Fig. S22A, B). However, the ferroptosis pathway showed suppressed activity in A549-R (Fig. 6A). Proteomic data analysis of A549-R and A549 xenograft tumor models showed significant differences in protein expression profiles in the two groups (Fig. S23A). A total of 479 DEPs were identified (Fig. 6B, Fig. S23B). These DEPs were enriched in iron ion homeostasis and ion binding (Fig. 6C), as well as drug metabolism pathways (Fig. 6D). Meanwhile, A549-R and A549 xenograft tumor models showed consistent differences in drug response (Fig. 6E) and also altered iron homeostasis (Fig. 6F). Additionally, other pathways related to apoptosis, oxygen response, and oxidative stress were also affected (Fig. S24A-C).

Notably, as a critical upstream regulator of HO-1, the Nrf2 pathway was significantly enriched (Fig. 6G) and activated in the A549-R xenograft tumor models (Fig. 6H). Additional pathway enrichment results were shown in Table S4. It is reported that Nrf2 plays a pivotal role in drug resistance and ferroptosis regulation [26,27]. Our results also found that Nrf2 pathway, including P62, Nrf2, and HO-1, were activated in A549-R. While the ferroptosis-inhibiting protein GPX4 was also significantly upregulated in A549-R, confirming the suppression of ferroptosis in A549-R cells (Fig. 6I, J). Further experiments revealed that activation of the Nrf2 pathway might be induced by cisplatin, as evidenced by the fact that low doses of cisplatin promoted the expression of Nrf2 pathway and down-regulated the expression of Keap1 (Fig. 6K, L). These results strongly support the crucial role of the Nrf2/HO-1 axis in mediating cisplatin resistance development (Fig. 6M). Inhibition of the downstream key protein HO-1 in this pathway may represent a potential strategy to overcome drug resistance.

### SB 202190 and NDGA reverse cisplatin resistance by inhibiting HO-1 and reactivating ferroptosis

**Fig. 7:**
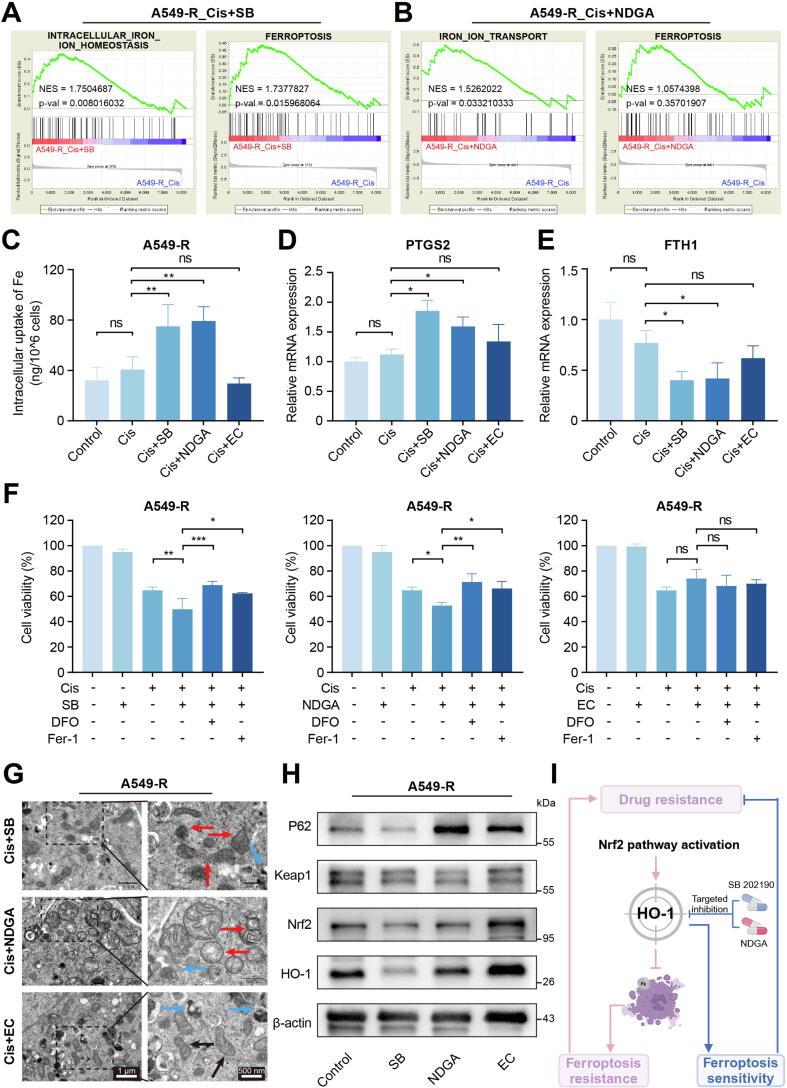
SB 202190 and NDGA inhibited the expression of HO-1, thereby activating ferroptosis. GSEA analysis of (A) cisplatin + SB 202190 and (B) cisplatin + NDGA. (C) Concentrations of intracellular Fe after cisplatin and the combination of cisplatin and candidate compounds treatment. Ferroptosis biomarkers, including (D) PTGS2 and (E) FTH1 were detected. (F) Fer-1 and DFO inhibited the cell death induced by the combination of candidate compounds and cisplatin. (G) Representative TEM images of different group. The black arrows indicate normal mitochondria, the red arrows indicate the abnormal mitochondria, and the blue arrows indicate the apoptotic body. (H) Western blotting showed that SB 202190 and NDGA inhibited the expression of HO-1, but had no effect on Nrf2, P62 and Keap1. (I) Schematic diagram of the mechanism by which the addition of SB 202190 and NDGA restores cellular sensitivity to cisplatin. All experiments were independently repeated at least three times. (For interpretation of the references to colour in this figure legend, the reader is referred to the web version of this article.)

Given the critical role of ferroptosis in cisplatin resistance in NSCLC, further investigation was conducted to determine whether SB 202190 and NDGA overcome cisplatin resistance by reactivating the ferroptosis pathway. Proteomic data analysis for these candidate compounds were performed, and DEPs, GO and KEGG enrichment analysis were presented in Fig. S25-S27. GSEA analysis demonstrated that SB 202190 combined with cisplatin significantly affected the drug response (Fig. S28A), suppressing autophagy (Fig. S28B) without notably affecting apoptosis or necroptosis (Fig. S28C). Additionally, the upregulation of ROS biosynthesis pathways and the downregulation of ROS metabolic pathways suggested that the combination of SB 202190 and cisplatin may lead to ROS accumulation (Fig. S28D). Meanwhile, SB 202190 significantly activated the ferroptosis pathway and influenced iron homeostasis (Fig. 7A). NDGA combined with cisplatin suppressed autophagy (Fig. S29A), but had no significant impact on apoptosis or necroptosis pathways (Fig. S29B). NDGA influenced iron homeostasis, but it did not appear to activate ferroptosis pathways to the same extent as SB 202190 (Fig. 7B). EC did not show significant modulation of iron homeostasis or ferroptosis pathways (Fig. S30).

ICP-MS confirmed that both SB 202190 and NDGA combined with cisplatin increased intracellular iron levels in A549-R cells (Fig. 7C). Furthermore, the combination of SB 202190 and NDGA with cisplatin restored cisplatin's regulatory effects on PTGS2 and FTH1 expression in A549-R cells (Fig. 7D, E). Additionally, the regulation of GPX4 and SLC7A11 by the combination of SB 202190 and NDGA with cisplatin is shown in Fig. S31. Further experiments with DFO and Fer-1 showed they could inhibit the cell death induced by the combination of candidate compounds and cisplatin (Fig. 7F). The cisplatin + SB 202190 and cisplatin + NDGA group exhibited morphological characteristics of ferroptosis, including irregular mitochondrial morphology, increased vacuolization, mitochondrial membrane collapse, and a reduction in mitochondrial cristae (Fig. 7G). To investigate the contribution of other modes of cell death in the combination of candidate compounds with cisplatin, apoptosis inhibitors (Z-VAD-FMK), necroptosis inhibitors (Necrostatin-1), or autophagy inhibitors (3-Methyladenine) were used in combination with cisplatin/cisplatin + candidate compounds. It was found that these inhibitors could not rescue the cell death induced by cisplatin/cisplatin + candidate compounds (Fig. S32A-C).

Further experimental results showed that SB 202190 and NDGA inhibited the expression of HO-1, but did not affect the upstream protein Nrf2, Keap1 and P62 (Fig. 7H), indicating that SB 202190 and NDGA targeted HO-1 to block the Nrf2-mediated ferroptosis inhibition. To exclude the potential influence of off-target effects of the candidate compounds, we further knocked down HMOX1 in the A549-R cell line to explore the sustained effects of the combination. The results showed that the A549-R exhibited increased sensitivity to cisplatin after HMOX1 knockdown, which is consistent with previous results. Additionally, both DFO and Fer-1 demonstrated a rescue effect on cell death, indicating that the inhibition of HMOX1 restored the cells' sensitivity to ferroptosis (Fig. S33A). The effects of the candidate compounds in combination with cisplatin were validated in A549-R with HMOX1-knockdown. It was found that the drug combination did not exhibit a more significant cytotoxic effect compared to cisplatin alone, indicating that the sensitizing effect of SB 202190 and NDGA on cisplatin was influenced by the down-expression of HMOX1 (Fig. S33B). Crucially, the synergistic interaction between SB 202190/NDGA and cisplatin was inhibited in HMOX1 knockdown cells, providing definitive evidence of HMOX1′s central role in mediating this combinatorial therapeutic effect (Fig. S34A, B). These findings suggest that cisplatin-induced ferroptosis was suppressed in A549-R, and the combination of SB 202190 or NDGA could reactivate ferroptosis by inhibiting HO-1, thereby restoring cellular sensitivity to cisplatin (Fig. 7I).

## Discussion

Although emerging drugs and therapies are rapidly developing [28], including immunotherapy, targeted therapy, etc., which have quickly entered the clinic [29], traditional chemotherapy and chemotherapy combinations remain the main treatment strategies in the clinic [30]. Among them, platinum drugs, as the most widely used broad-spectrum antitumor drugs [31], and will continue to be extensively used in the foreseeable future. However, drug resistance to platinum drugs remains a widespread issue [32], which often leads to worse outcomes in NSCLC patients treated with platinum chemoterapy.

There are some studies on platinum drug resistance, including the exploration of resistance genes and targeted strategies [33,34], nanoparticle-based interventions [35], combination strategies with immunotherapy [6], targeted exosome strategies [36] and so on. However, none have achieved substantial breakthroughs. The limited clinical translation of these strategies arises primarily from insufficient large-scale cohort studies to systematically investigate platinum resistance mechanisms [37]. Although molecular biology is rapidly developing, there is still a lack of widely recognized resistant cell models, patient-derived tumor xenograft models and patient-derived tumour organoids [38], especially since the evolution of resistance in cell models is stochastic, making it difficult for different teams to achieve consistent results in their resistance models [39]. In addition, the mechanisms of platinum resistance and the role of platinum drugs in the drug resistance process are still unclear. Despite advances in multi-omics platforms, there is still a lack of sufficient sequencing information to help elucidate the molecular mechanisms underlying the development of resistance. For instance, in NSCLC research, so far, only a few datasets, including GSE108214 and GSE84146, contain the transcriptome data of cisplatin sensitive and resistant cells of common NSCLC cell lines, and more information, including proteomics, is still lacking, which also hinders the development of platinum drug resistance-based solutions. Safety concerns surrounding these drug resistance-overcoming strategies, including the toxicity of combination therapies and side effects linked to immunotherapy regimens, have also drawn persistent attention. Consequently, a core challenge demanding urgent resolution lies in further validating resistance mechanisms and identifying more precise resistance-related targets to screen safer potential drugs.

Based on the above problems, this study established cisplatin resistance cell lines using the widely employed A549 NSCLC cell line as the parental line, followed by transcriptomic profiling to identify potential resistance mechanisms and candidate genes. Through integrative analysis combining these data with publicly available transcriptomic datasets of additional cisplatin resistant/sensitive A549 cell lines from GEO, along with other cisplatin resistant/sensitive NSCLC models (H23 and H460), HMOX1 was consistently identified as being significantly upregulated across multiple resistant cell lines. This study further analyzed the TCGA database to find that high expression of HMOX1 is associated with poor patient outcomes (overall survival, disease specific survival), suggesting the correlational role of HMOX1 in platinum resistance and poor patient outcomes. HO-1, a critical protein involved in intracellular oxygen sensing and oxidative stress regulation, exerts cytoprotective mechanisms—including antioxidant, anti-apoptotic, and anti-inflammatory properties—to rescue cells from death [[40], [41], [42]]. Cancer cells exploit this by upregulating HO-1 and its mediated antioxidant defenses to counteract oxidative stress, thereby attenuating the efficacy of anticancer therapies [43]. Moreover, such adaptive responses further enhance cellular tolerance to stressors, ultimately leading to the development of therapeutic resistance [44]. In summary, the transcriptomic data from various drug resistant cell models, along with the key role of HMOX1 in the development of resistance and its clinical relevance, demonstrate that HMOX1 is a potential cisplatin resistance gene with significant research value.

In further exploration of resistance mechanisms, joint analysis of proteomics and transcriptomics revealed ferroptosis resistance in cisplatin resistance cells, with the Nrf2/HO-1 pathway, being significantly enriched. Through systematic experimental validation, we revealed that low-dose cisplatin exposure induces the activation of the Nrf2 signaling pathway in resistant cell lines. Mechanistically, as the upstream regulator of HO-1, Nrf2-mediated drug resistance have been extensively reported in multiple cancer types. It is reported that the activation of Nrf2-ARE pathway induced the artesunate resistance in head and neck cancer cells, while inhibition of the Nrf2 pathway reduced ferroptosis resistance [20]. Some studies have highlighted Nrf2-based interventions as potential strategies to overcome drug resistance. For example, lncRNA mir4435-2HG has been shown to modulate cisplatin resistance in HCT116 cells through the Nrf2/HO-1 pathway [45], and similar findings have been reported for miR-29b-3p [46]. Additionally, GULP1, a Keap1-binding protein, has been reported to regulate Keap1-Nrf2 signaling and affect drug sensitivity in urothelial carcinoma [47]. Given that the Nrf2-ARE pathway regulates multiple downstream genes (including HMOX1, FTH1, SLC7A11, and GPX4) involved in iron metabolism, intermediary metabolism, and GSH synthesis/metabolism [26], therapeutic targeting of this pathway presents considerable challenges for overcoming chemoresistance. Consequently, pharmacological inhibition of HO-1, the key downstream effector of Nrf2 signaling, emerges as a potentially viable strategy to circumvent this clinically significant resistance mechanism.

In order to explore the mechanism of HMOX1-mediated drug resistance and its potential value as a therapeutic target, we screened drugs based on HMOX1 translation protein (HO-1) to further explore possible solutions to reverse platinum drug resistance. Among the candidates, SB 202190 and NDGA emerged as promising agents warranting further investigation and therapeutic exploration. SB 202190, an inhibitor of p38 MAPK [48], has been explored for its broad pharmacological effects, including modulation of mitochondrial Ca^2+^ concentrations [49]. Mailem RC et al. identified SB 202190 as a potential immune-targeting therapeutic for acute respiratory distress syndrome, systemic inflammatory response syndrome, sepsis and COVID-19 [50]. However, its potential role in overcoming platinum-based drug resistance has not been previously reported. NDGA, a compound with decades of application, has been shown to have therapeutic value in a wide range of conditions, including cancer treatment [51], neurological disorders [52], cardiovascular diseases [53], and arthritis [54]. NDGA has also been reported to play a role in NSCLC progression, metastasis, and chemoresensitization [55]. However, its potential application in overcoming platinum-based resistance in NSCLC and the underlying mechanisms remain unclear.

Notably, both SB 202190 and NDGA have not been reported to have significant toxic side effects. SB 202190 has been shown to improve postoperative outcomes in glaucoma filtration surgery [56]. Tang et al. further demonstrated its pharmacological potential in mitigating extracellular matrix accumulation and fibrosis in diabetic nephropathy [57]. In animal studies, SB 202190 was found to alleviate muscle atrophy while prolonging survival in tumor-bearing mice [58]. Similarly, NDGA has displayed neuroprotective effects by reducing neuroinflammation and ischemic injury in murine models [52]. Given the severe toxicity of cisplatin, Mundhe et al. specifically evaluated NDGA's impact on cisplatin-induced nephrotoxicity. Their results indicated that NDGA pretreatment not only avoided exacerbating oxidative stress, nitrosative stress, or inflammation but also conferred renal protection by restoring IL-10 levels and preserving renal function [59]. Consistent with these findings, our *in vivo* experiments revealed no additional adverse effects on organ function, blood biochemical indexes, or behavioral outcomes in mice. These collective data underscore the safety profiles of SB 202190 and NDGA, highlighting their potential as adjuvant therapies to cisplatin.

Through *in vitro* and *in vivo* experiments and mechanistic exploration, we conclusively demonstrated that both NDGA and SB 202190 target HO-1 to reverse cisplatin resistance. Mechanistically, these compounds inhibit the Nrf2-mediated ferroptosis resistance pathway via HO-1 suppression, thereby restoring cellular susceptibility to ferroptosis and synergizing with cisplatin to enhance therapeutic efficacy. These findings underscore that the Nrf2/HO-1 pathway serves as a pivotal mediator of cisplatin-induced ferroptosis resistance, thereby driving chemoresistance. This highlights the significance of HO-1 as both a biomarker for platinum resistance and a therapeutic target for resistance reversal.

Notably, while our study, leveraging multi-omics data and experimental validation, delineated the role of HMOX1 in cisplatin resistance and identified SB 202190 and NDGA as promising agents to counteract drug resistance, several limitations warrant caution. Firstly, the absence of long-term toxicity assessments raises concerns about the clinical translatability of SB 202190 and NDGA. Secondly, the broader applicability of HO-1-targeted therapies across diverse tumor types remains unexplored. Further investigations are imperative to validate the centrality of HO-1 in cisplatin resistance and to generalize its targeting strategy in pan-cancer analyses.

## Conclusion

In summary, this study established A549 cisplatin-resistant cell lines and analyzed their transcriptomic profiles to demonstrate HMOX1′s role in cisplatin resistance, which was further validated using GEO and TCGA datasets. Both *in vitro* and *in vivo* experiments confirmed HMOX1 as a key mediator of cisplatin resistance. Moreover, HO-1 intervention with NDGA/SB 202190 modulated intracellular iron homeostasis and restored ferroptosis by suppressing the Nrf2/HO-1 pathway, effectively reversing cisplatin resistance. This study systematically elucidates cisplatin resistance mechanisms in NSCLC, identifies HMOX1 as a crucial resistance gene, and identifies potential therapeutic interventions, offering novel strategies to overcome cisplatin resistance and improve clinical outcomes.

## Availability of data and materials

The datasets analyzed for this study can be found in the TCGA project (https://portal.gdc.cancer.gov/) and GEO database (https://www.ncbi.nlm.nih.gov/geo/). The other data relevant to the study are included in the article or uploaded as online supplementary information.

## Compliance with Ethics Requirements

All animal experimental protocols were reviewed and approved by the Institutional Animal Care and Use Committee, Wenzhou Institute, University of Chinese Academy of Sciences (No. WIUCAS23011701).

## Declaration of competing interest

The authors declare that they have no known competing financial interests or personal relationships that could have appeared to influence the work reported in this paper.
