# Supplementary material for: Heme oxygenase 1 (HO-1) is a drug target for reversing cisplatin resistance in non-small cell lung cancer
Source: J Adv Res. 2025 May 17;80:1121–37. doi: 10.1016/j.jare.2025.05.033 (PMC12869221; doi:10.1016/j.jare.2025.05.033)
Supplement: Supplementary Data 1 [file mmc1.pdf]

Supplementary Tables

Heme Oxygenase 1 (HO-1) is a drug target for reversing cisplatin resistance in non-small cell lung cancer

Table S1. Primers of each gene.

Gene	Forward	Reverse
ACTB	5'-CCTGGCACCCAGCACAAT-3'	5'-GGGCCGGACTCGTCATAC-3'
HMOX1	5'-TGCCAGTGCCACCAAGTTCAAG-3'	5'-TGTTGAGCAGGAACGCAGTCTTG-3'
Nrf2	5'-AGTCCAGAAGCCAAACTGACAGAAG-3'	5'-GGAGAGGATGCTGCTGAAGGAATC-3'
GPX4	5'-CCGCTGTGGAAGTGGATGAAGATC-3'	5'-CTTGTCGATGAGGAACTGTGGAGAG-3'
Keap1	5'-GCTGTCCTCAATCGTCTCCTTTATG-3'	5'-TCATTCGCCACTCGTTCCTCTC-3'
P62	5'-AGTCGGATAACTGTTCAGGAGGAG-3'	5'-TCGGATTCTGGCATCTGTAGGG-3'
PTGS2	5'-GGGTTGCTGGTGGTAGGAATGTTC-3'	5'-CTGGTATTTCATCTGCCTGCTCTGG-3'
FTH1	5'-CCGCCATCAACCGCCAGATC-3'	5'-CAGTTTCTCAGCATGTTCCCTCTCC-3'
SLC7A11	5'-ACGGTGGTGTGTTTGCTGTCTC-3'	5'-GCTGGTAGAGGAGTGTGCTTGC-3'
PPARGC 1A	5'-CACGGACAGAACTGAGGGAC-3'	5'-TTCGTTTGACCTGCGCAAAG-3'
APOBEC 3F	5'-ATAGACCCATCCTTTCTCGTCG-3'	5'-CCTTGAGGGACCCTTTGTTTT-3'
ASGR1	5'-ATGAAGTCGCTAGAGTCCCAG-3'	5'-CAGGTCAGACACGAACTGCTT-3'
ANGPTL 4	5'-GTCCACCGACCTCCCGTTA-3'	5'-CCTCATGGTCTAGGTGCTTGT-3'
FURIN	5'-GAGCCCAAAGACATCGGGAA-3'	5'-GCCACGGCGATTATAGGACA-3'
PCSK9	5'-CCTGGAGCGGATTACCCCT-3'	5'-CTGTATGCTGGTGTCTAGGAGA-3'
CDA	5'-GGCAATTGCTATCGCCAGTG-3'	5'-TCCGGCTTGGTCATGTACAC-3'
APOH	5'-ATACAATTACCTGCACGACACAT-3'	5'-GGCCATCCAGAGAATATCCATCA-3'
A1CF	5'-TGTGGACAACTGCCGATTATTT-3'	5'-TGACATCGACAACACCTTCAGTA-3'

Table S2. Basic information of the three candidate compounds in animal experiments.

Drugs	Administration Concentration	Administration Method	Administration Frequencies	Solvent	Storage
SB 202190	0.05mg/kg	Intraperitoneal injection	Once every 3 days for 8 times	Dissolved in DMSO and diluted with saline	-20°C
NDGA	40 mg/kg	Intraperitoneal injection	Once every 3 days for 8 times	Dissolved in DMSO and diluted with saline	-20°C
EC	5mg/kg	Intraperitoneal injection	Once every 3 days for 8 times	0.5% CMC-Na	Freshly prepared

Table S3. Results of docking with HO-1 and basic information of drugs.

Item	Catalog_ NO	Drug_Name	Docking _score	CAS_No.	Formula	Mw
1	HY-N0198	Nordihydroguaiaretic acid	-12.682	500-38-9	$C_{18}H_{22}O_4$	302.36
2	HY-18963	Lavendustin A	-12.362	125697-92-9	$C_{21}H_{19}NO_6$	381.38
3	HY-18965	TAS-301	-12.171	193620-69-8	$C_{23}H_{19}NO_3$	357.40
4	HY-16751	Ralinepag	-11.862	1187856-49-0	$C_{23}H_{26}ClNO_5$	431.91
5	HY-16680	Helioxanthin 8-1	-11.816	840529-13-7	$C_{20}H_{12}N_2O_6$	376.32
6	HY-122312	BAY-8002	-11.609	724440-27-1	$C_{20}H_{14}ClNO_5S$	415.85
7	HY-13413	Tofogliflozin (hydrate)	-11.514	1201913-82-7	$C_{22}H_{28}O_7$	386.44
8	HY-13226	Galunisertib	-11.226	700874-72-2	$C_{22}H_{19}N_5O$	369.42
9	HY-N0001	(-)-Epicatechin	-11.221	490-46-0	$C_{15}H_{14}O_6$	290.27
10	HY-N0116	Hematoxylin	-11.188	517-28-2	$C_{16}H_{14}O_6$	302.28
11	HY-15613	Maritoclax	-11.087	1227962-62-0	$C_{22}H_{12}Cl_4N_2O_4$	510.15
12	HY-100881	TUG-891	-11.021	1374516-07-0	$C_{23}H_{21}FO_3$	364.41
13	HY-12242	GBR 12935 (dihydrochloride)	-10.971	67469-81-2	$C_{28}H_{36}Cl_2N_2O$	414.58
14	HY-17465	Glycopyrrolate	-10.966	596-51-0	$C_{19}H_{28}BrNO_3$	318.43
15	HY-B1132	Clidinium (bromide)	-10.954	3485-62-9	$C_{22}H_{26}BrNO_3$	352.45
16	HY-14544	Quetiapine	-10.840	111974-69-7	$C_{21}H_{25}N_3O_2S$	383.51
17	HY-N0072	Brazilin	-10.835	474-07-7	$C_{16}H_{14}O_5$	286.28
18	HY-14927	Lificiguat	-10.811	170632-47-0	$C_{19}H_{16}N_2O_2$	304.34
19	HY-B2070	Methylbenactyzium Bromide	-10.780	3166-62-9	$C_{21}H_{28}BrNO_3$	342.45
20	HY-N0225	(-)-Epigallocatechin	-10.712	970-74-1	$C_{15}H_{14}O_7$	306.27
21	HY-100140	Pipequaline	-10.653	77472-98-1	$C_{22}H_{24}N_2$	316.44
22	HY-107139	JNJ-10229570	-10.604	524923-88-4	$C_{22}H_{19}N_3O_2S$	389.47
23	HY-112055	DIM-C-pPhOH	-10.510	151358-47-3	$C_{23}H_{18}N_2O$	338.40
24	HY-13501	Mubritinib	-10.465	366017-09-6	$C_{25}H_{23}F_3N_4O_2$	468.47
25	HY-B0984	Fendiline (hydrochloride)	-10.418	13636-18-5	$C_{23}H_{26}ClN$	315.45
26	HY-B0962	Piperidolate (hydrochloride)	-10.347	129-77-1	$C_{21}H_{26}ClNO_2$	323.43
27	HY-10111	TG100-115	-10.344	677297-51-7	$C_{18}H_{14}N_6O_2$	346.34
28	HY-B2098	Lucanthone	-10.315	479-50-5	$C_{20}H_{24}N_2OS$	340.48
29	HY-N0162	Luteolin	-10.305	491-70-3	$C_{15}H_{10}O_6$	286.24

(Continues)

Table S3 (Continued)

Item	Catalog_ NO	Drug_Name	Docking _score	CAS_No.	Formula	Mw
30	HY-B0808	Oxaprozin	-10.300	21256-18-8	$C_{18}H_{15}NO_3$	293.32
31	HY-I0230	Solifenacin (hydrochloride)	-10.270	180468-39-7	$C_{23}H_{27}ClN_2O_2$	362.46
32	HY-A0002	Solifenacin (Succinate)	-10.270	242478-38-2	$C_{27}H_{32}N_2O_6$	362.46
33	HY-A0087	Octocrylene	-10.264	6197-30-4	$C_{24}H_{27}NO_2$	361.48
34	HY-103683	PF-06409577	-10.174	1467057-23-3	$C_{19}H_{16}ClNO_3$	341.79
35	HY-101419	CYM-5541	-10.169	945128-26-7	$C_{19}H_{28}N_2O_2$	316.44
36	HY-11035	WAY-262611	-10.163	1123231-07-1	$C_{20}H_{22}N_4$	318.42
37	HY-111492	DIM-C-pPhOCH3	-10.097	33985-68-1	$C_{24}H_{20}N_2O$	352.43
38	HY-N0637	Eriodictyol	-10.096	552-58-9	$C_{15}H_{12}O_6$	288.25
39	HY-12239	CID755673	-10.089	521937-07-5	$C_{12}H_{11}NO_3$	217.22
40	HY-N0112	Dihydromyricetin	-10.088	27200-12-0	$C_{15}H_{12}O_8$	320.25
41	HY-19370	FPS-ZM1	-10.034	945714-67-0	$C_{20}H_{22}ClNO$	327.85
42	HY-18296	AKT-IN-1	-10.031	1357158-81-6	$C_{22}H_{21}N_3O$	343.42
43	HY-19808	C-DIM12	-9.989	178946-89-9	$C_{23}H_{17}ClN_2$	356.85
44	HY-N6628	6,2'-Dihydroxyflavone	-9.951	92439-20-8	$C_{15}H_{10}O_4$	254.24
45	HY-N6771	Cyclopiazonic acid	-9.941	18172-33-3	$C_{20}H_{20}N_2O_3$	336.38
46	HY-103710	IBR2	-9.927	313526-24-8	$C_{24}H_{20}N_2O_2S$	400.49
47	HY-100852	ER-000444793	-9.919	792957-74-5	$C_{23}H_{18}N_2O_2$	354.40
48	HY-12589	ZM39923 (hydrochloride)	-9.895	1021868-92-7	$C_{23}H_{26}ClNO$	331.45
49	HY-B1135	Benzbromarone	-9.890	3562-84-3	$C_{17}H_{12}Br_2O_3$	424.08
50	HY-15204	Tonabersat	-9.888	175013-84-0	$C_{20}H_{19}ClFNO_4$	391.82
51	HY-19958	XEN907	-9.878	912656-34-9	$C_{21}H_{21}NO_4$	351.40
52	HY-114166	2-D08	-9.873	144707-18-6	$C_{15}H_{10}O_5$	270.24
53	HY-B0887	Permethrin	-9.834	52645-53-1	$C_{21}H_{20}Cl_2O_3$	391.29
54	HY-10004	Faropenem daloxate	-9.826	141702-36-5	$C_{17}H_{19}NO_8S$	397.40
55	HY-B1090	Cinnarizine	-9.809	298-57-7	$C_{26}H_{28}N_2$	368.51
56	HY-N0125	Diosmetin	-9.796	520-34-3	$C_{16}H_{12}O_6$	300.26
57	HY-108708	GeA-69	-9.790	2143475-98-1	$C_{20}H_{16}N_2O$	300.35
58	HY-18787	c-Fms-IN-2	-9.769	791587-67-2	$C_{19}H_{21}N_3O_3$	339.39
59	HY-10110	IC-87114	-9.756	371242-69-2	$C_{22}H_{19}N_7O$	397.43

(Continues)

Table S3 (Continued)

Item	Catalog_NO	Drug_Name	Docking_score	CAS_No.	Formula	Mw
60	HY-B0970	Diphenylpyraline (hydrochloride)	-9.752	132-18-3	$C_{19}H_{24}ClNO$	281.39
61	HY-B0230	Phenylbutazone	-9.735	50-33-9	$C_{19}H_{20}N_2O_2$	308.37
62	HY-15394	(Rac)-Rotigotine (hydrochloride)	-9.685	102120-99-0	$C_{19}H_{26}ClNOS$	315.47
63	HY-N0182	Fisetin	-9.647	528-48-3	$C_{15}H_{10}O_6$	286.24
64	HY-N0003	Honokiol	-9.616	35354-74-6	$C_{18}H_{18}O_2$	266.33
65	HY-N0109	Salidroside	-9.610	10338-51-9	$C_{14}H_{20}O_7$	300.30
66	HY-12059	AT7867	-9.607	857531-00-1	$C_{20}H_{20}ClN_3$	337.85
67	HY-A0007	Rotigotine (Hydrochloride)	-9.590	125572-93-2	$C_{19}H_{26}ClNOS$	315.47
68	HY-100201	A-196	-9.588	1982372-88-2	$C_{18}H_{16}Cl_2N_4$	359.25
69	HY-12243	CID-797718	-9.586	370586-05-3	$C_{12}H_{11}NO_3$	217.22
70	HY-107390	AX-024	-9.583	1370544-73-2	$C_{21}H_{22}FNO_2$	339.40
71	HY-10115	PI-103	-9.580	371935-74-9	$C_{19}H_{16}N_4O_3$	348.36
72	HY-19805	STO-609	-9.521	52029-86-4	$C_{19}H_{10}N_2O_3$	314.29
73	HY-10295	SB 202190	-9.513	152121-30-7	$C_{20}H_{14}FN_3O$	331.34
74	HY-W013372	7,8-Dihydroxyflavone	-9.500	38183-03-8	$C_{15}H_{10}O_4$	254.24
75	HY-11068	SB 239063	-9.493	193551-21-2	$C_{20}H_{21}FN_4O_2$	368.40
76	HY-N0168	Hesperetin	-9.479	520-33-2	$C_{16}H_{14}O_6$	302.28
77	HY-B1143	Broxaldine	-9.472	3684-46-6	$C_{17}H_{11}Br_2NO_2$	421.08
78	HY-101462	RAD51 Inhibitor B02	-9.382	1290541-46-6	$C_{22}H_{17}N_3O$	339.39
79	HY-16560	Camptothecin	-9.378	7689-03-4	$C_{20}H_{16}N_2O_4$	348.35
80	HY-B0979	Lobeline (hydrochloride)	-9.342	134-63-4	$C_{22}H_{28}ClNO_2$	337.46
81	HY-19625	MCB-613	-9.340	1162656-22-5	$C_{20}H_{20}N_2O$	304.39
82	HY-103039	TLR7 agonist 2	-9.327	1642857-69-9	$C_{17}H_{16}N_6O_2$	336.35
83	HY-115502	BCI	-9.310	1245792-51-1	$C_{22}H_{23}NO$	317.42
84	HY-18008	PS-1145	-9.277	431898-65-6	$C_{17}H_{11}ClN_4O$	322.75
85	HY-15945	DBeQ	-9.272	177355-84-9	$C_{22}H_{20}N_4$	340.42
86	HY-125833	Alpha-Naphthoflavone	-9.237	604-59-1	$C_{19}H_{12}O_2$	272.30
87	HY-B0740	Cyclobenzaprine (hydrochloride)	-9.206	6202-23-9	$C_{20}H_{22}ClN$	275.39

(Continues)

Table S3 (Continued)

Item	Catalog_ NO	Drug_Name	Docking _score	CAS_No.	Formula	Mw
88	HY-B0198	Cefaclor	-9.170	53994-73-3	$C_{15}H_{14}ClN_3O_4S$	367.81
89	HY-13953	JIB-04	-9.140	199596-05-9	$C_{17}H_{13}ClN_4$	308.76
90	HY-B1880	Oxadiazon	-9.138	19666-30-9	$C_{15}H_{18}Cl_2N_2O_3$	345.22
91	HY-15574	Piboserod	-9.111	152811-62-6	$C_{22}H_{31}N_3O_2$	369.50
92	HY-12043	SB 525334	-9.094	356559-20-1	$C_{21}H_{21}N_5$	343.42
93	HY-112051	CU-CPT9b	-9.091	2162962-69-6	$C_{16}H_{13}NO_2$	251.28
94	HY-15762	Valdecoxib	-9.057	181695-72-7	$C_{16}H_{14}N_2O_3S$	314.36
95	HY-10591	Neuropathiazol	-9.035	880090-88-0	$C_{19}H_{18}N_2O_2S$	338.42
96	HY-122198	ML367	-9.034	381168-77-0	$C_{19}H_{12}F_2N_4$	334.32
97	HY-42110	Deschloroclozapine	-9.012	1977-07-7	$C_{18}H_{20}N_4$	292.38
98	HY-114698	Retro-2 cycl	-8.996	1429192-00-6	$C_{19}H_{16}N_2OS$	320.41
99	HY-114181	IRAK4-IN-4	-8.970	1850276-58-2	$C_{22}H_{16}N_2O_2$	340.37
100	HY-100996	10074-G5	-8.962	413611-93-5	$C_{18}H_{12}N_4O_3$	332.31

Table S4. Pathway enrichment results of differentially expressed proteins.

ID	Term	List Hits	p-value	q-value	Enrichment_score
WP5390	Pancreatic cancer subtypes	9	7.634e-06	0.003611	6.482143
WP383	Striated muscle contraction pathway	6	0.000698	0.117513	5.458647
WP3644	NAD metabolism	4	0.000944	0.117513	8.642857
WP229	Irinotecan pathway	6	0.001058	0.117513	5.059233
WP2884	NRF2 pathway	12	0.001242	0.117513	2.784276
WP697	Estrogen metabolism	4	0.001517	0.119591	7.68254
WP2882	Nuclear receptors meta pathway	19	0.002815	0.190213	2.014899
WP534	Glycolysis and gluconeogenesis	20	0.004438	0.227535	1.894325
WP545	Complement activation	4	0.004598	0.227535	5.761905
WP2645	Heroin metabolism	2	0.00481	0.227535	17.28571
WP69	T cell receptor signaling pathway	11	0.005487	0.235948	2.437729
WP262	Ebstein Barr virus LMP1 signaling	4	0.006183	0.243715	5.318681
WP2826	Cocaine metabolism	2	0.007865	0.248455	13.82857
WP558	Complement and coagulation cascades	6	0.00809	0.248455	3.400468
WP4721	Eicosanoid metabolism via lipooxygenases LOX	4	0.008095	0.248455	4.938776
WP1545	miRNAs involved in DNA damage response	3	0.008404	0.248455	6.914286
WP2291	Deregulation of Rab and Rab effector genes in bladder cancer	3	0.010126	0.269933	6.482143
WP5347	IL 26 signaling pathways	5	0.011074	0.269933	3.677812
WP364	IL6 signaling pathway	9	0.011205	0.269933	2.449944
WP692	Sulfation biotransformation reaction	3	0.012037	0.269933	6.10084
