# Supplementary material for: Heme oxygenase 1 (HO-1) is a drug target for reversing cisplatin resistance in non-small cell lung cancer
Source: J Adv Res. 2025 May 17;80:1121–37. doi: 10.1016/j.jare.2025.05.033 (PMC12869221; doi:10.1016/j.jare.2025.05.033)
Supplement: Supplementary Data 2 [file mmc2.pdf]

Supplementary Figures

Heme Oxygenase 1 (HO-1) is a drug target for reversing cisplatin resistance in non-small cell lung cancer

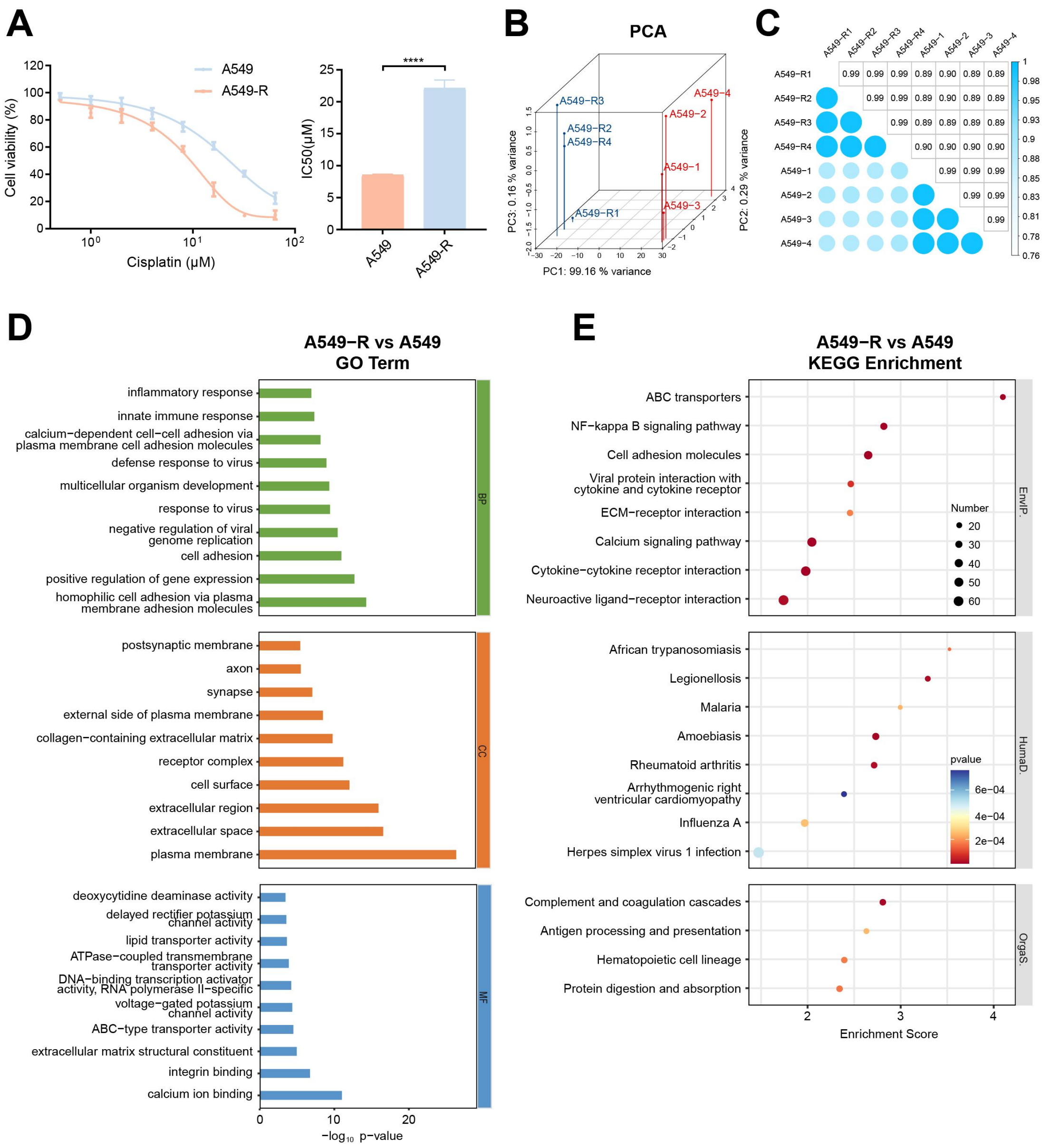

Figure S1. Transcriptome analysis of cisplatin-resistant NSCLC cell line A549-R and NSCLC cell line A549. (A) The survival rate of A549 and A549-R at the cisplatin administration concentration,

and the IC50 of A549 and A549-R. All experiments were independently repeated at least three times. (B) Principal component analysis (PCA) and (C) correlational box plot of transcriptome data. (D) GO and (E) KEGG enrichment analysis of differential expression genes (DEGs).

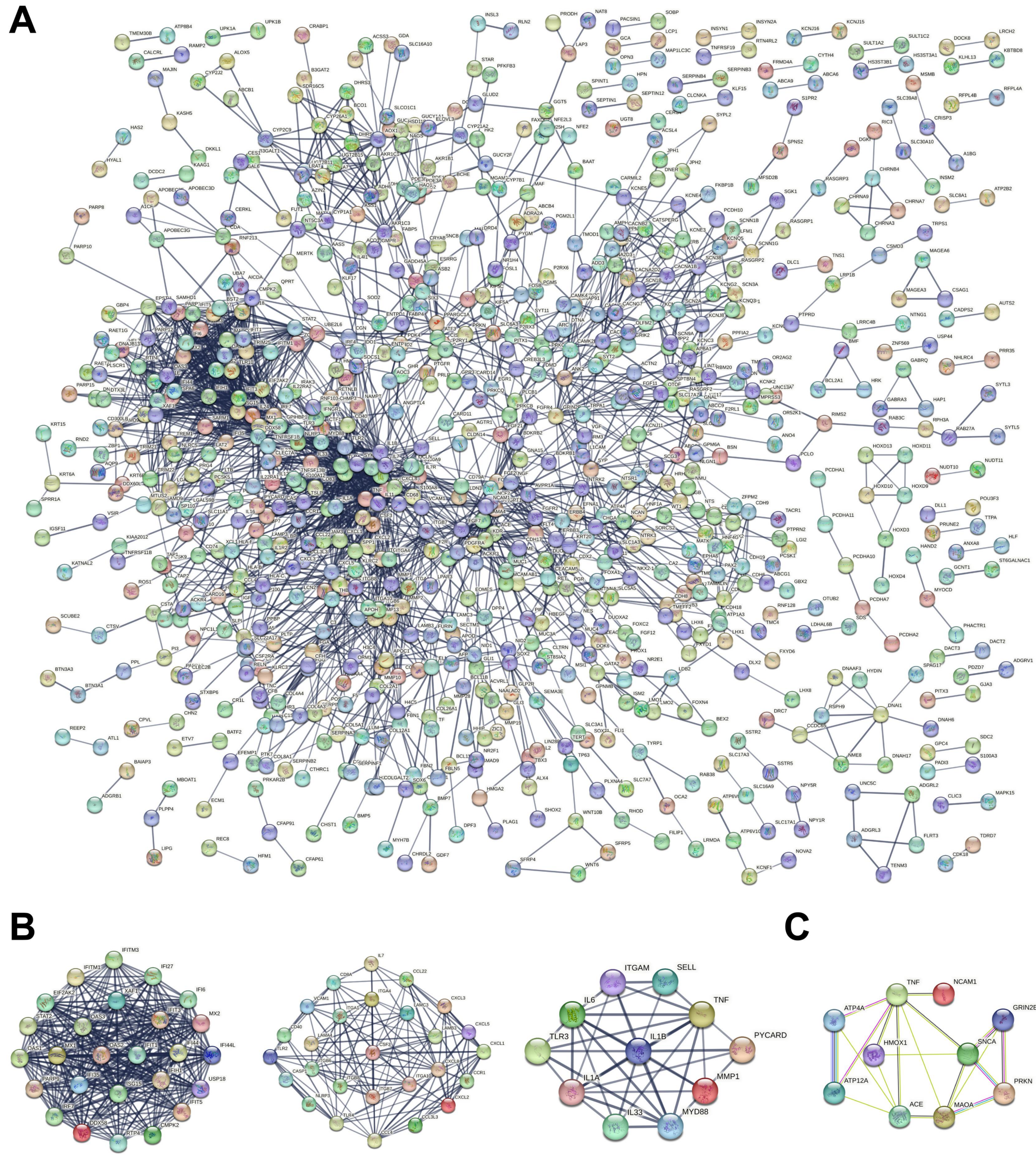

Figure S2. Protein-protein interaction (PPI) network analysis of DEGs (A549-R vs. A549). (A) PPI network of DEGs. (B) The core modules in the PPI network. (C) Top 10 hub genes were identified in PPI network.

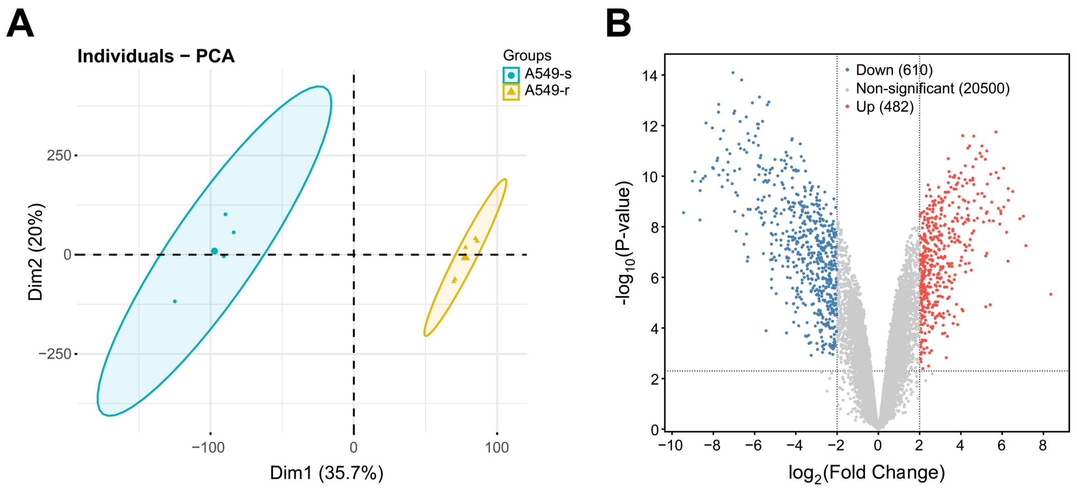

C

Category	ID	Term	Count	%	P-values
**GO functional enrichment analysis of DEGs**					
GO-BP	GO:0007411	axon guidance	12	4.8	3.58E-05
	GO:0043303	mast cell degranulation	5	2	1.91E-04
	GO:0048843	negative regulation of axon extension involved in axon guidance	5	2	3.00E-04
	GO:0003272	endocardial cushion formation	4	1.6	3.22E-04
	GO:0035556	intracellular signal transduction	15	6	6.00E-04
GO-CC	GO:0070062	extracellular exosome	58	23.2	8.44E-09
			103	41.2	1.13E-07
	GO:0009986	cell surface	25	10	7.00E-07
	GO:0005615	extracellular space	49	19.6	1.20E-06
	GO:0016324	apical plasma membrane	17	6.8	2.43E-05
GO-MF	GO:0005201	extracellular matrix structural constituent	8	3.2	6.71E-04
	GO:0005515	protein binding	179	71.6	0.0054418
	GO:1990782	protein tyrosine kinase binding	5	2	0.0070889
	GO:0070700	BMP receptor binding	3	1.2	0.0094333
	GO:0004713	protein tyrosine kinase activity	6	2.4	0.0098417
**KEGG pathway enrichment analysis of DEGs**					
KEGG	hsa05150	Staphylococcus aureus infection	7	2.8	0.0019628
	hsa04360	Axon guidance	9	3.6	0.0028374
	hsa05146	Amoebiasis	6	2.4	0.0112696
	hsa00330	Arginine and proline metabolism	4	1.6	0.0276086
	hsa04650	Natural killer cell mediated cytotoxicity	6	2.4	0.0279566

Figure S3. Transcriptome analysis of GSE108214 dataset. (A) PCA of GSE108214. (B) The volcano plot, (C) GO and KEGG enrichment analysis of DEGs in GSE108214.

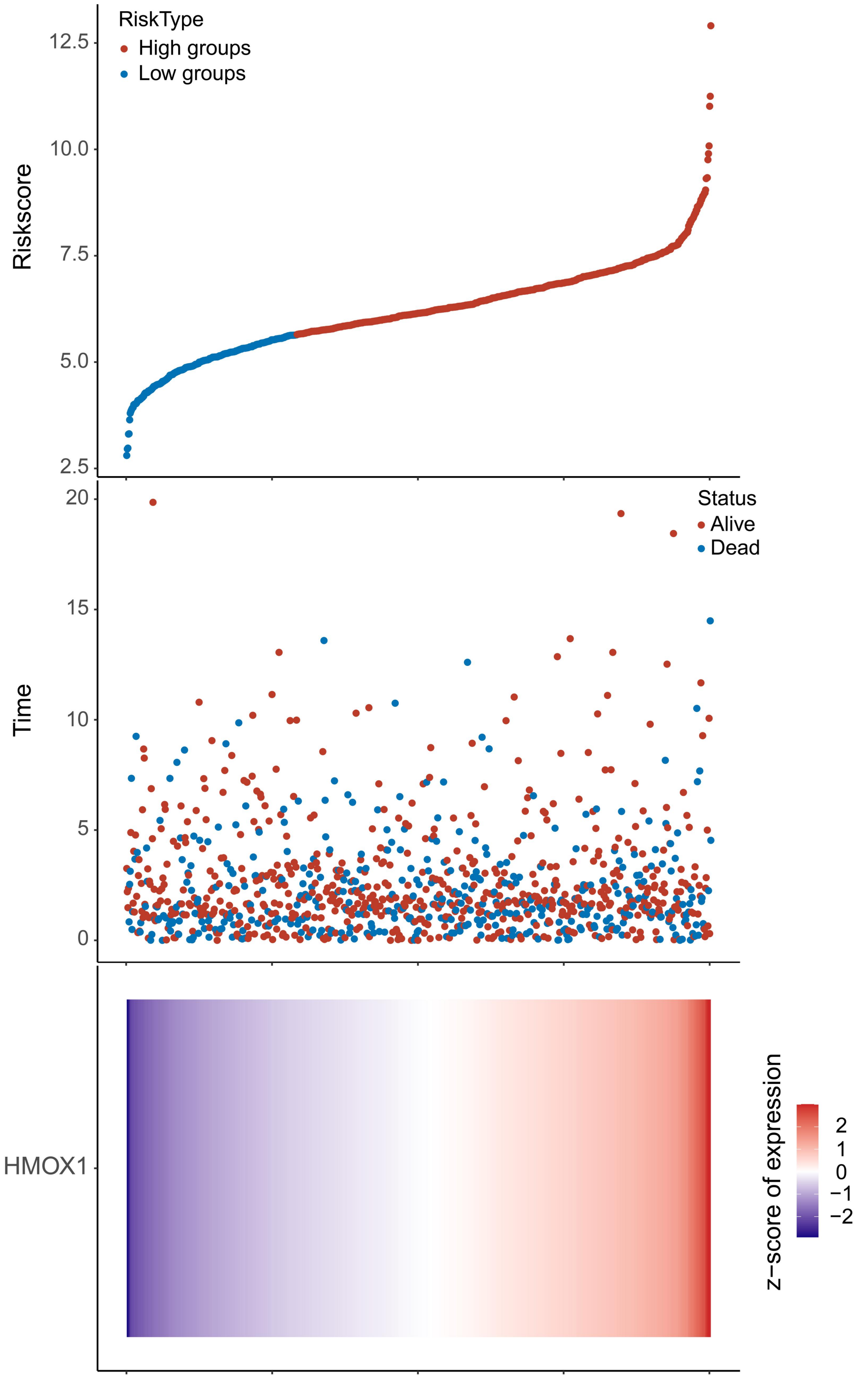

Figure S4. TCGA data showed that the expression of HMOX1 were negatively correlated with the prognosis of NSCLC patients.

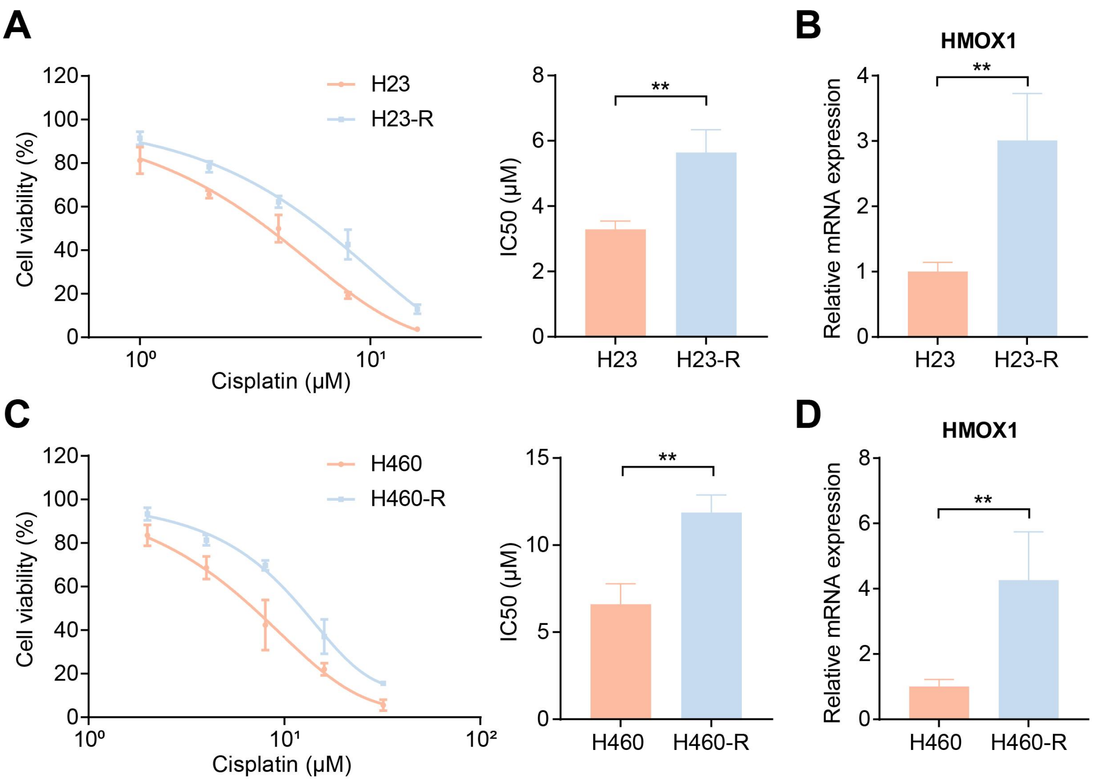

Figure S5. The validation of other NSCLC cell lines (H23, H460) and the corresponding cisplatin resistance cell lines (H23-R, H460-R). (A) The survival rate of H23 and H23-R at the cisplatin administration concentration, and the IC50 of H23 and H23-R. (B) RT-qPCR showed HMOX1 was upregulated in H23-R. (C) The survival rate of H460 and H460-R at the cisplatin administration concentration, and the IC50 of H460 and H460-R. (D) RT-qPCR showed HMOX1 was upregulated in H460-R. All experiments were independently repeated at least three times.

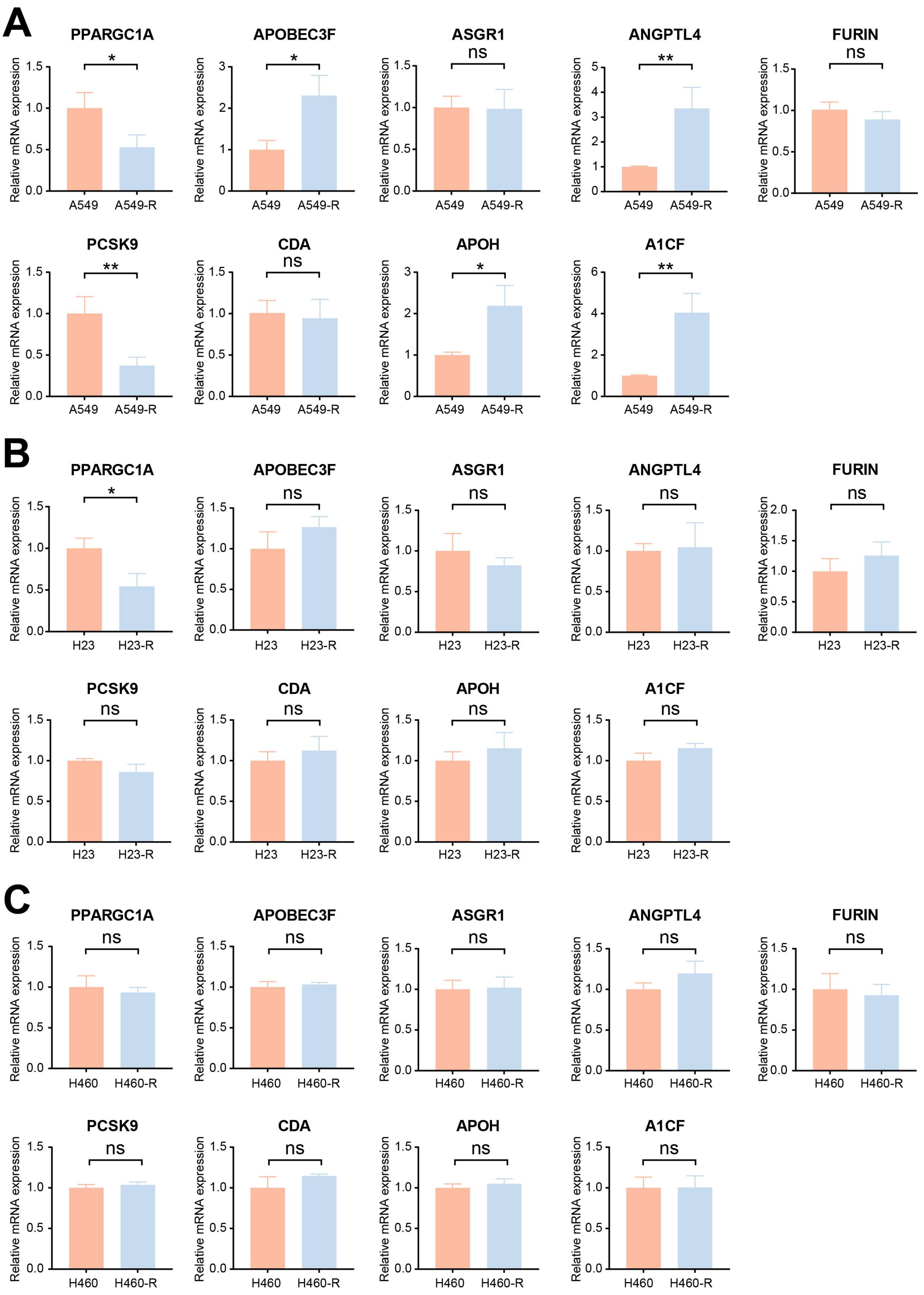

Figure S6. The expression of 10 core DEGs in self-established resistant cell lines and their sensitive cell lines. (A) A549 and A549-R, (B) H23 and H23-R, (C) H460 and H460-R.

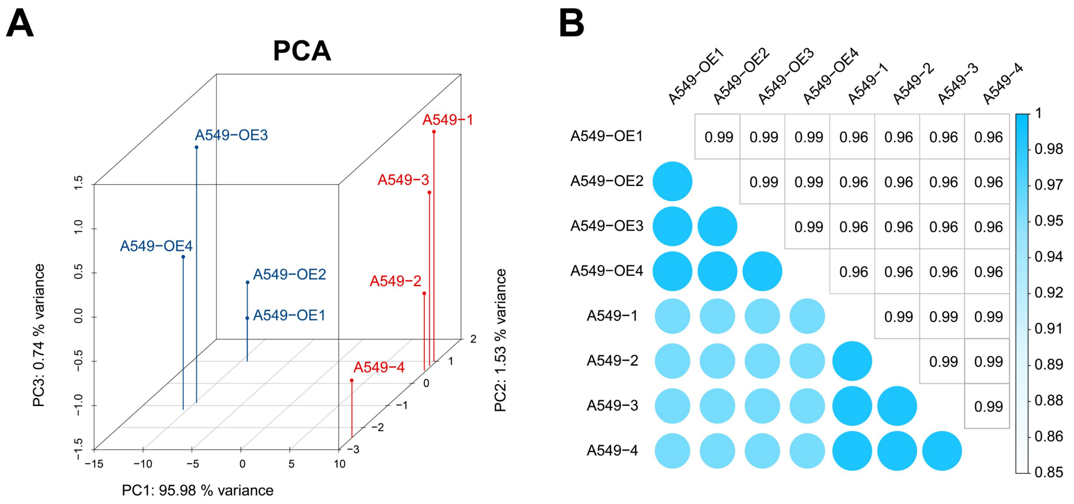

Figure S7. (A) PCA and (B) correlational box plot of transcriptome data of A549-HMOX1 and A549 cell lines.

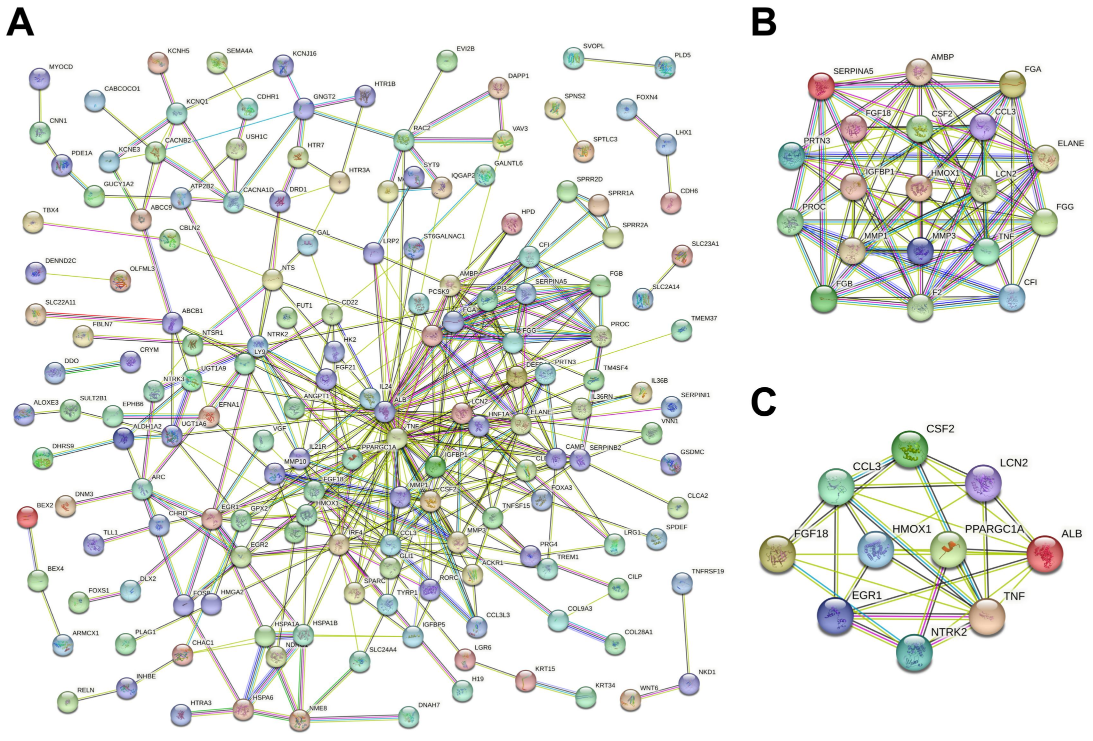

Figure S8. Protein-protein interaction (PPI) network analysis of DEGs (A549-HMOX1 vs. A549). (A) PPI network of DEGs. (B) The core module in the PPI network. (C) Top 10 hub genes were identified in PPI network.

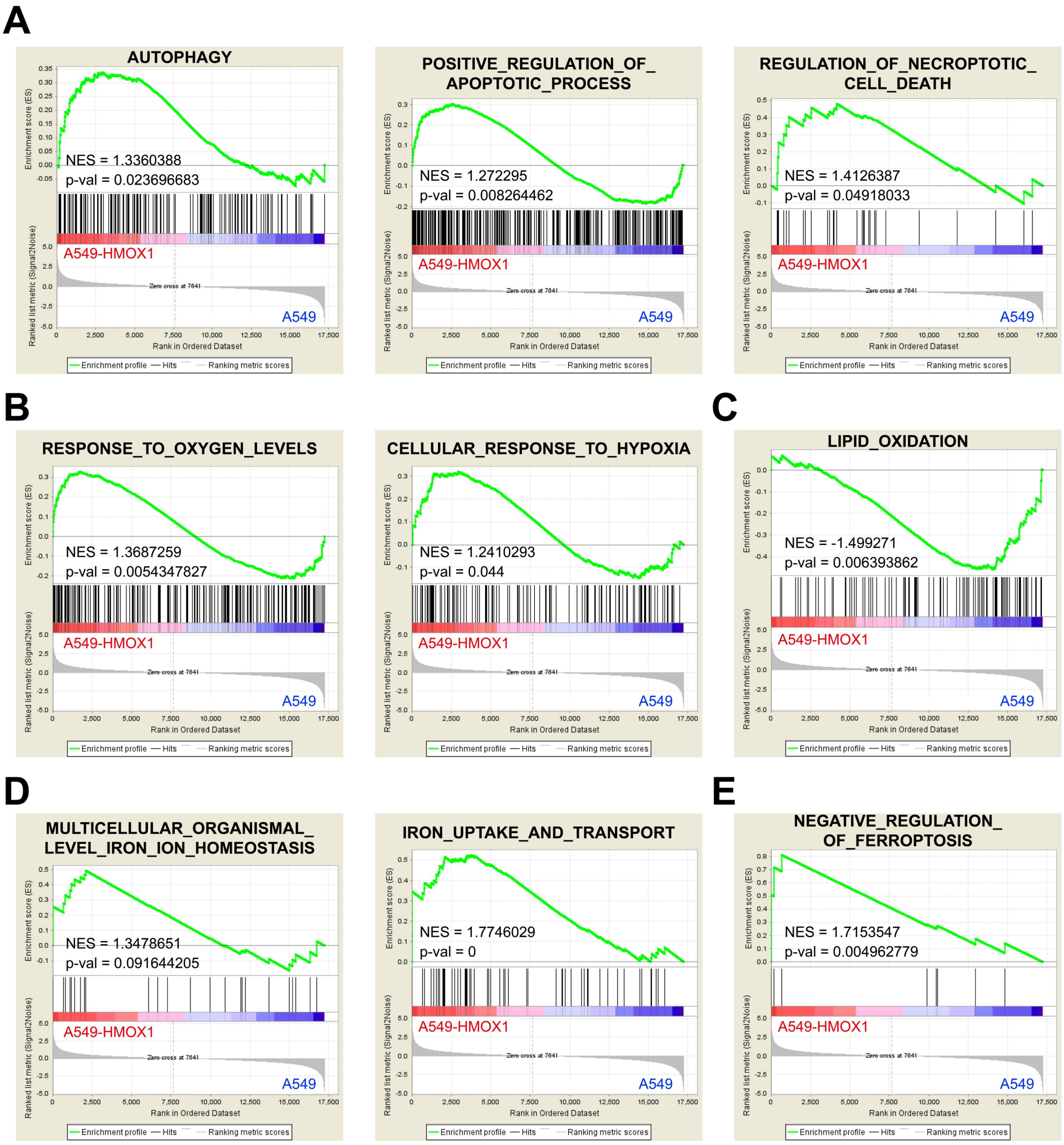

Figure S9. GSEA analysis of DEGs (A549-HMOX1 vs. A549). The overexpression of HMOX1 was positively correlated with (A) autophagy, apoptosis, necroptosis and (B) cellular response to oxygen. (C) The overexpression of HMOX1 is negatively correlated with lipid oxidation. The overexpression of HMOX1 was correlated with (D) iron ion homeostasis and (E) ferroptosis.

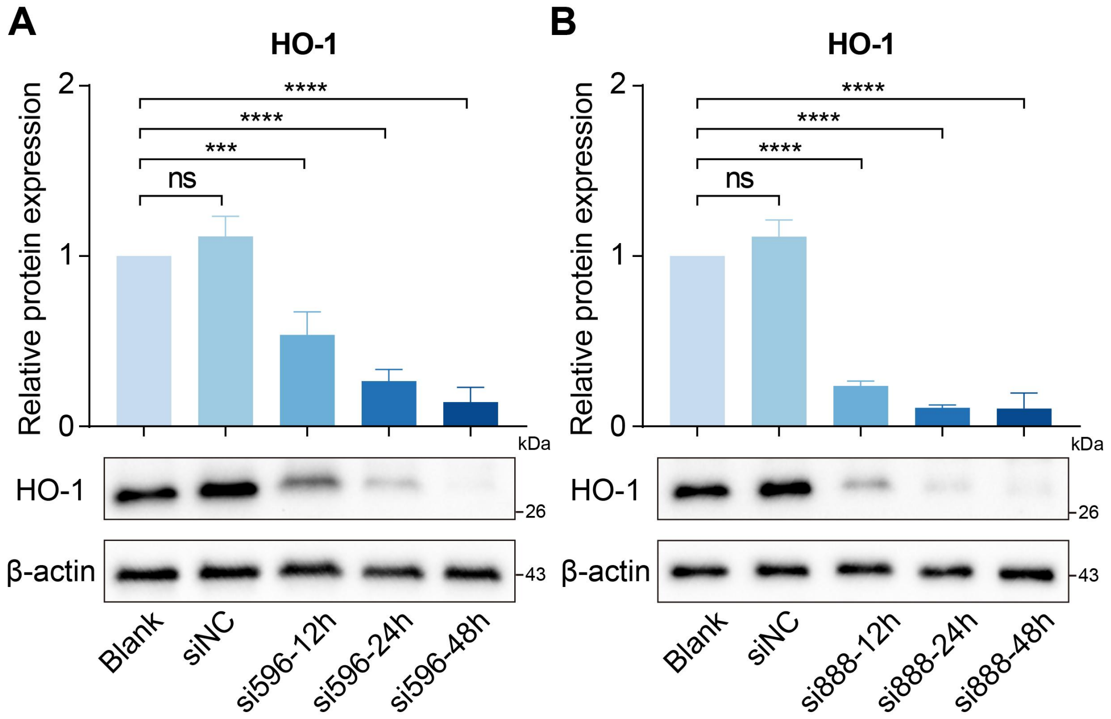

Figure S10. Effect of HMOX1 siRNA in A549-R cell. (A) si596 and (B) si888 can achieve excellent HMOX1-knockdown efficiency in 48h. All experiments were independently repeated at least three times.

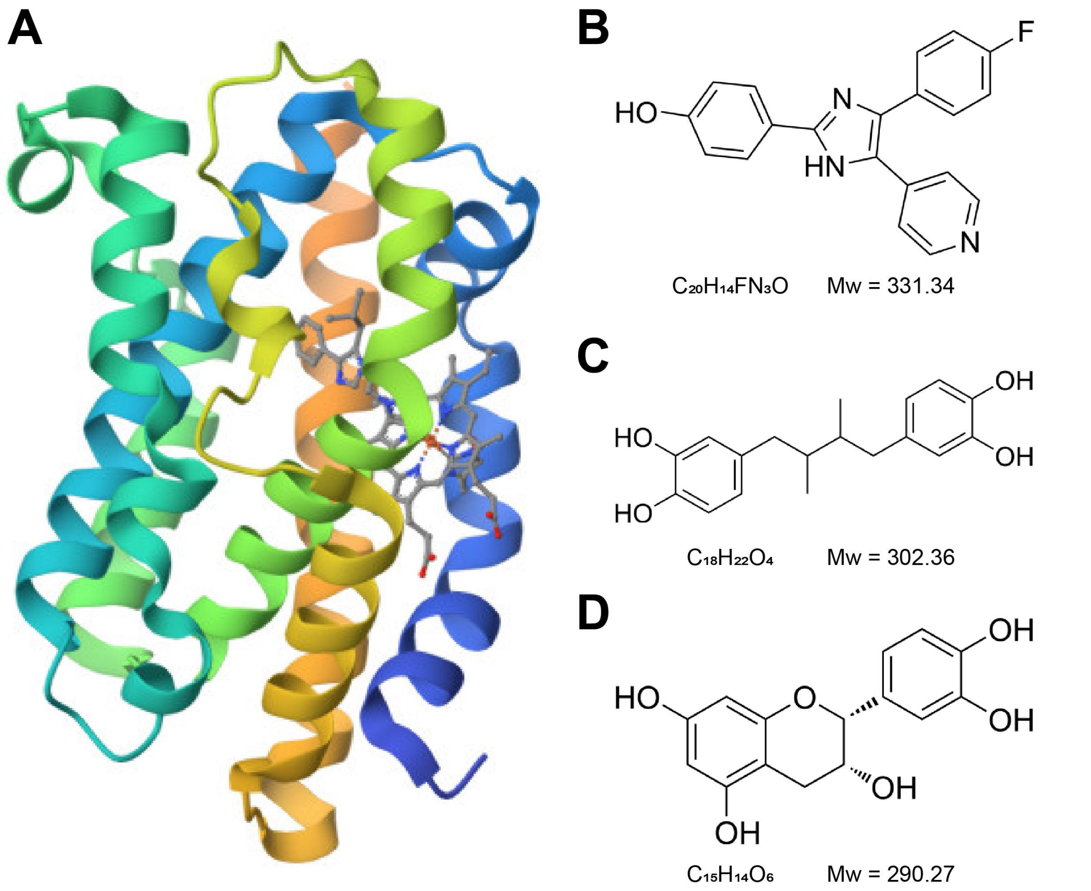

Figure S11. (A) The structure of HO-1 protein. The chemical structure of (B) SB 202190, (C) NDGA and (D) EC.

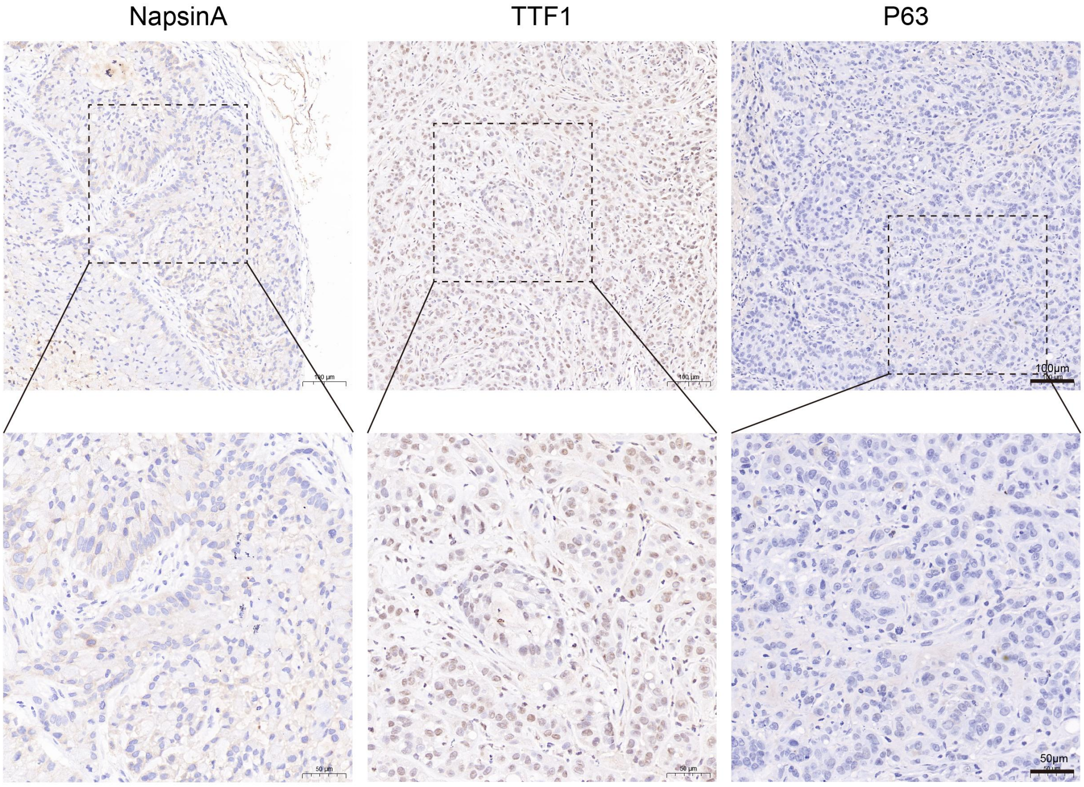

Figure S12. Representative IHC staining images of NSCLC's biomarkers in mice tumor. (n ≥ 3, scale bar = 100 μm or 50 μm).

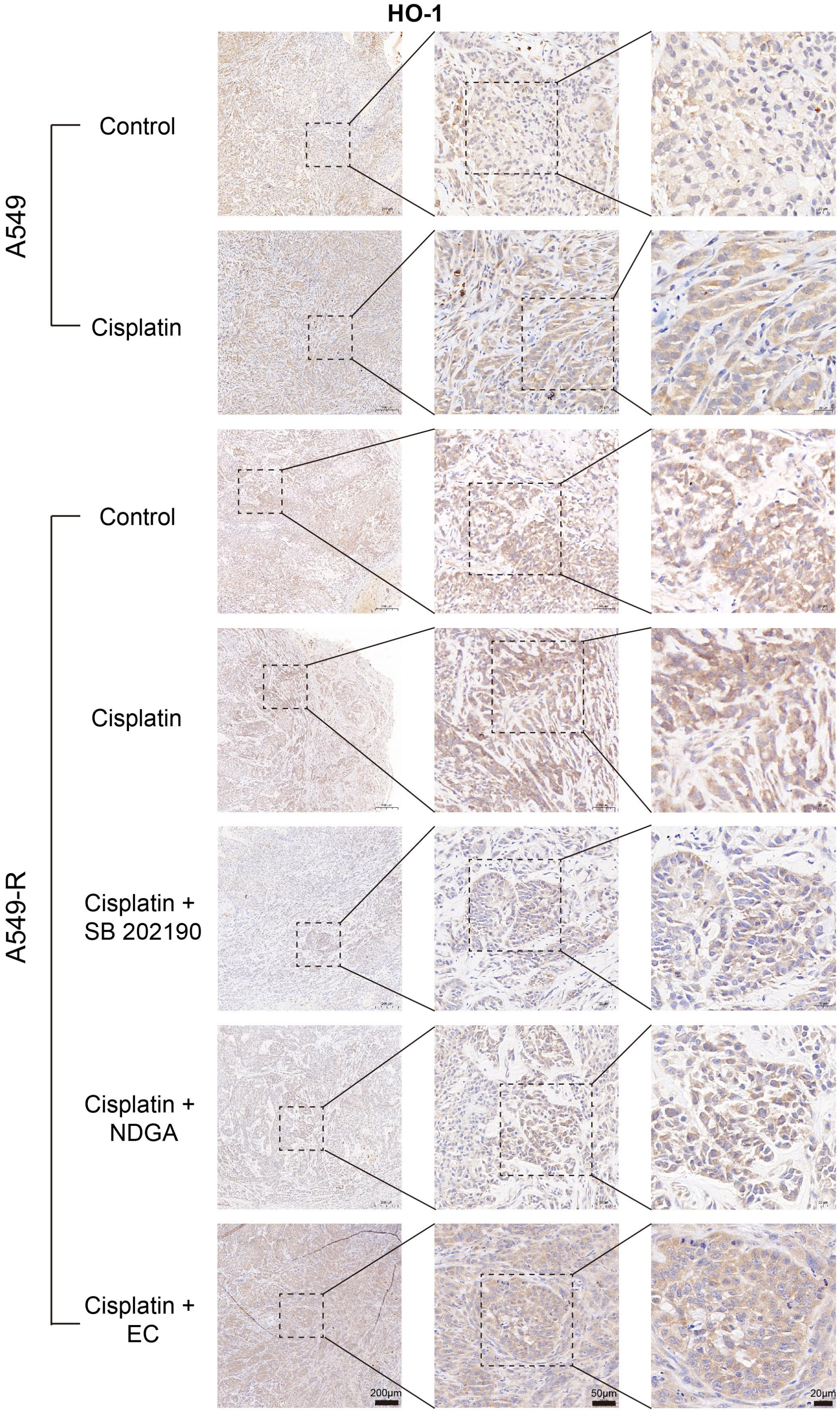

Figure S13. Representative IHC staining images of HO-1 in each group. (n ≥ 3, scale bar = 200 μm or 50 μm or 20 μm).

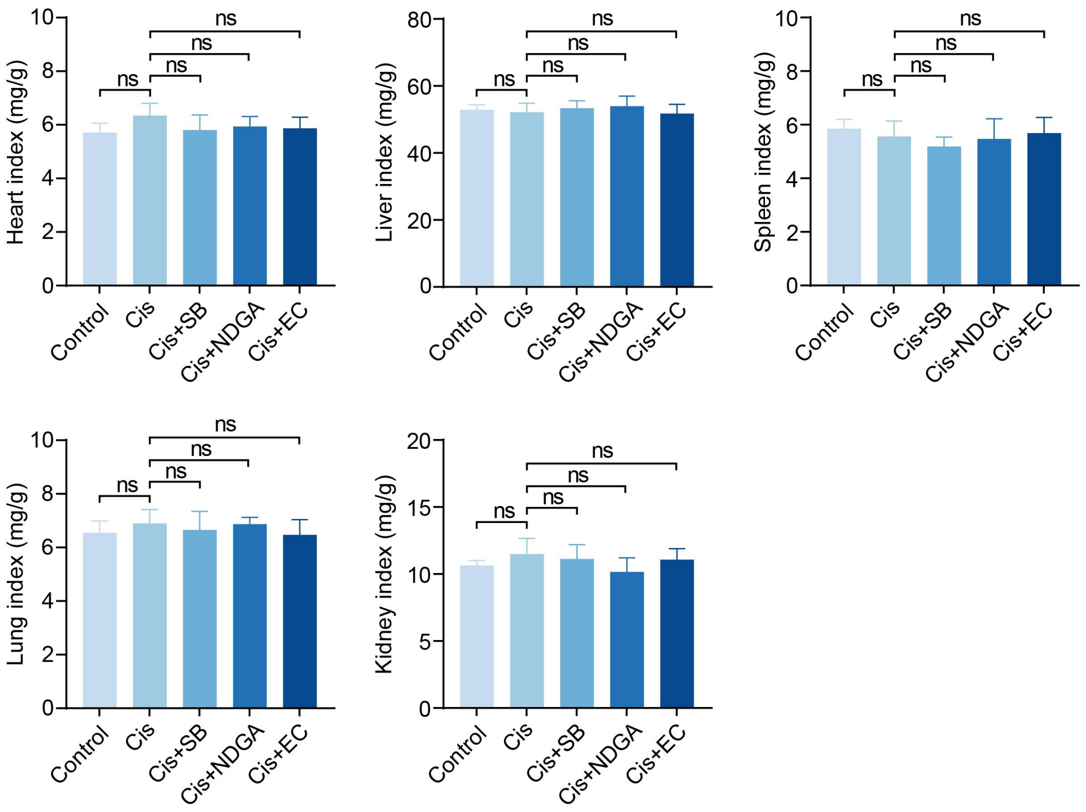

Figure S14. The indices of heart, liver, spleen, lung and kidney in each group of A549-R xenograft tumor models (n = 6 in each group).

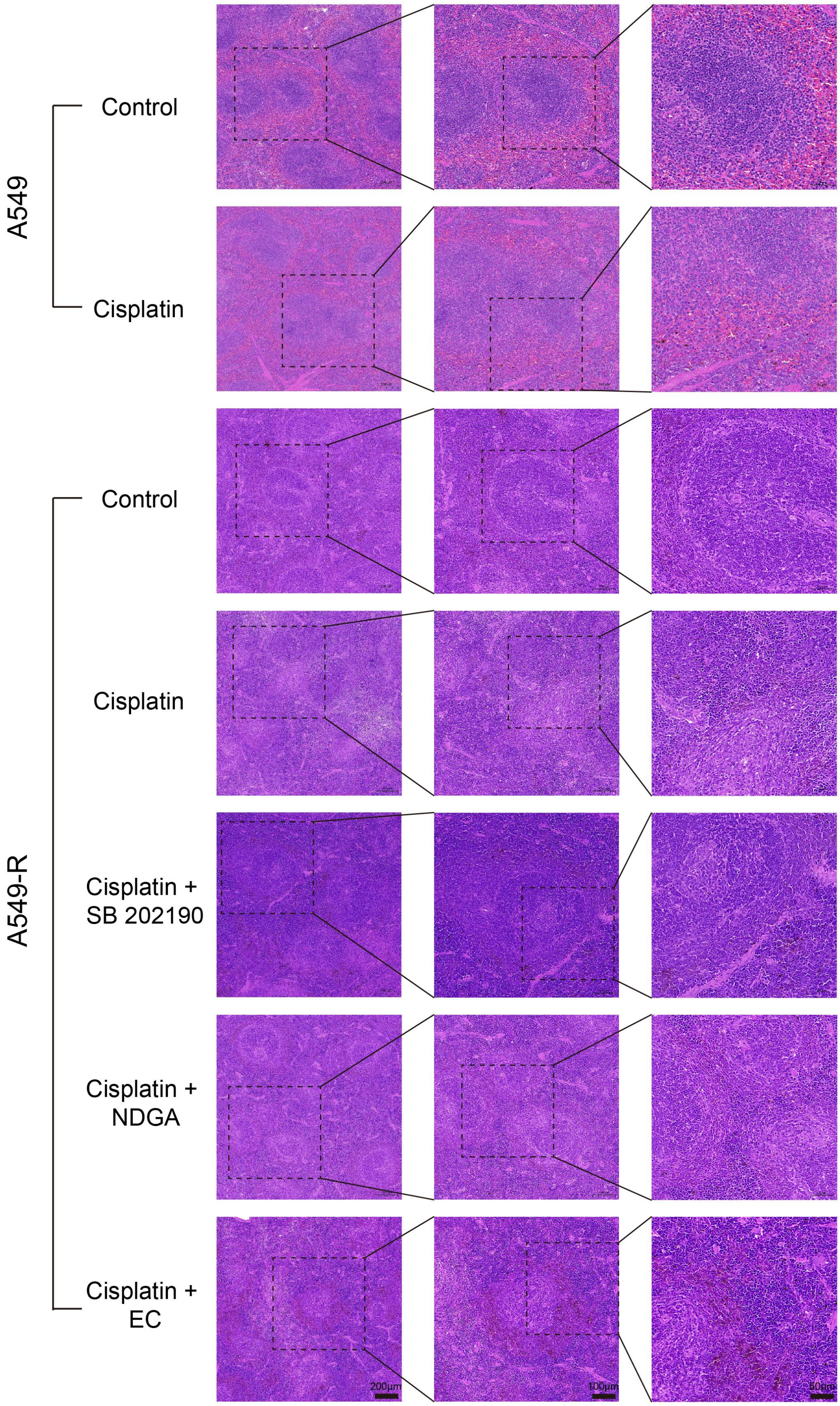

Figure S15. Representative H&E staining images of spleen tissues in each group. (n ≥ 3, scale bar = 200 μm or 100 μm or 50 μm).

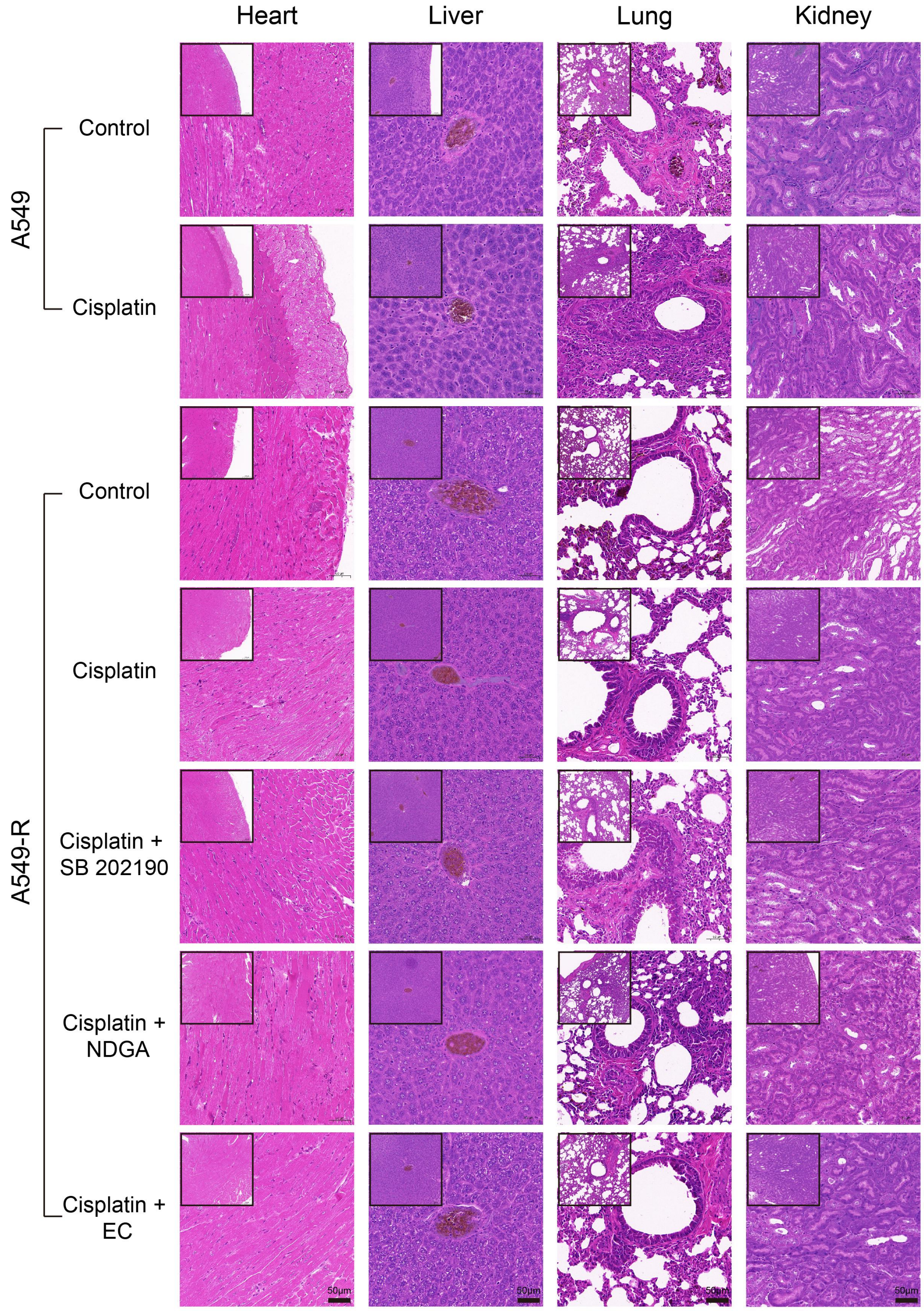

Figure S16. Representative H&E staining images of heart, liver, lung and kidney in each group. (n ≥ 3, scale bar = 100 μm or 50 μm).

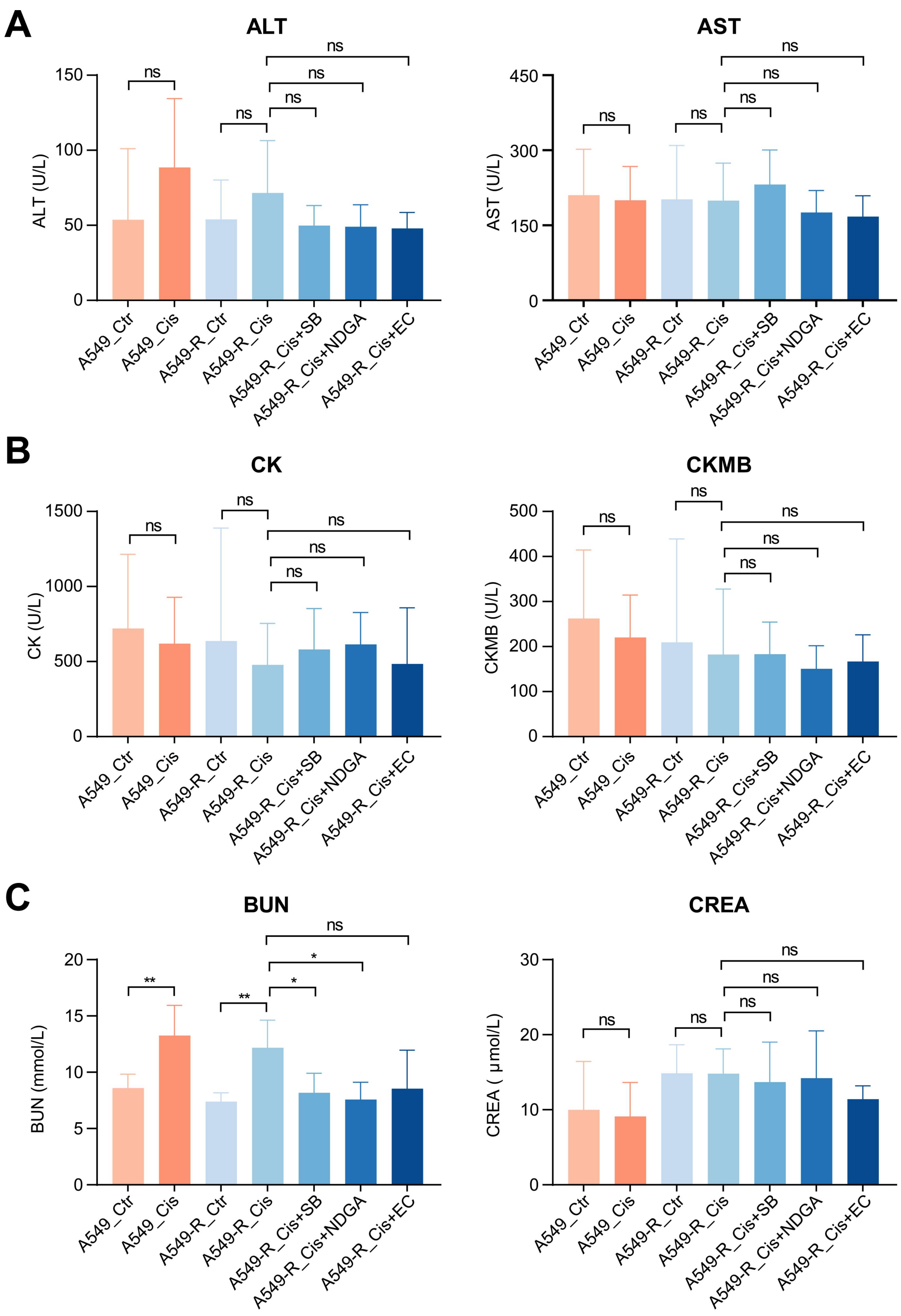

Figure S17. (A) ALT and AST levels (hepatic function), (B) CK and CKMB levels (cardiac function), (C) BUN and CREA levels (renal function) in serum of each group (n = 6 in each group).

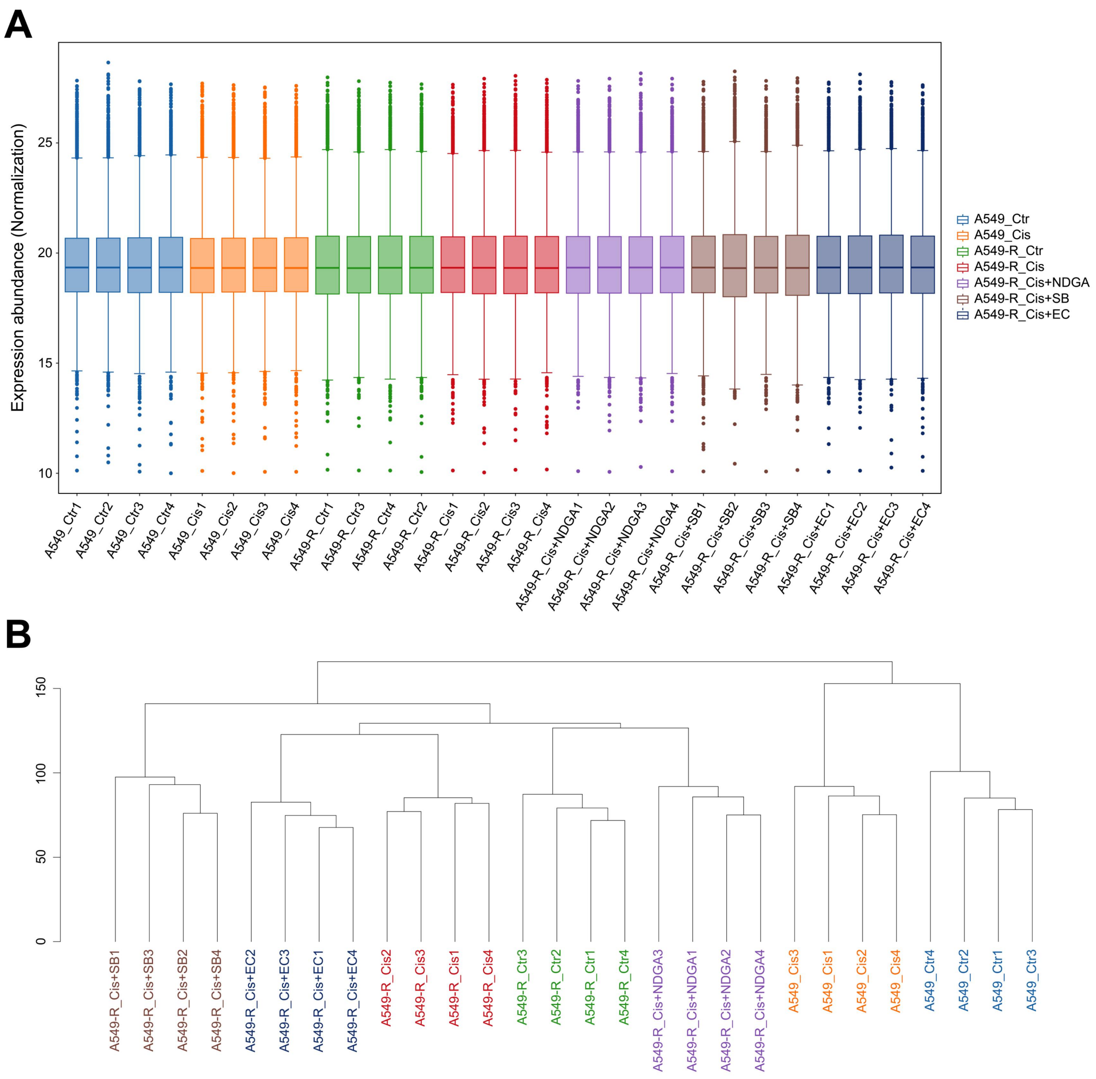

Figure S18. (A) The plot of sample expression abundance and (B) tree plot of proteomic data.

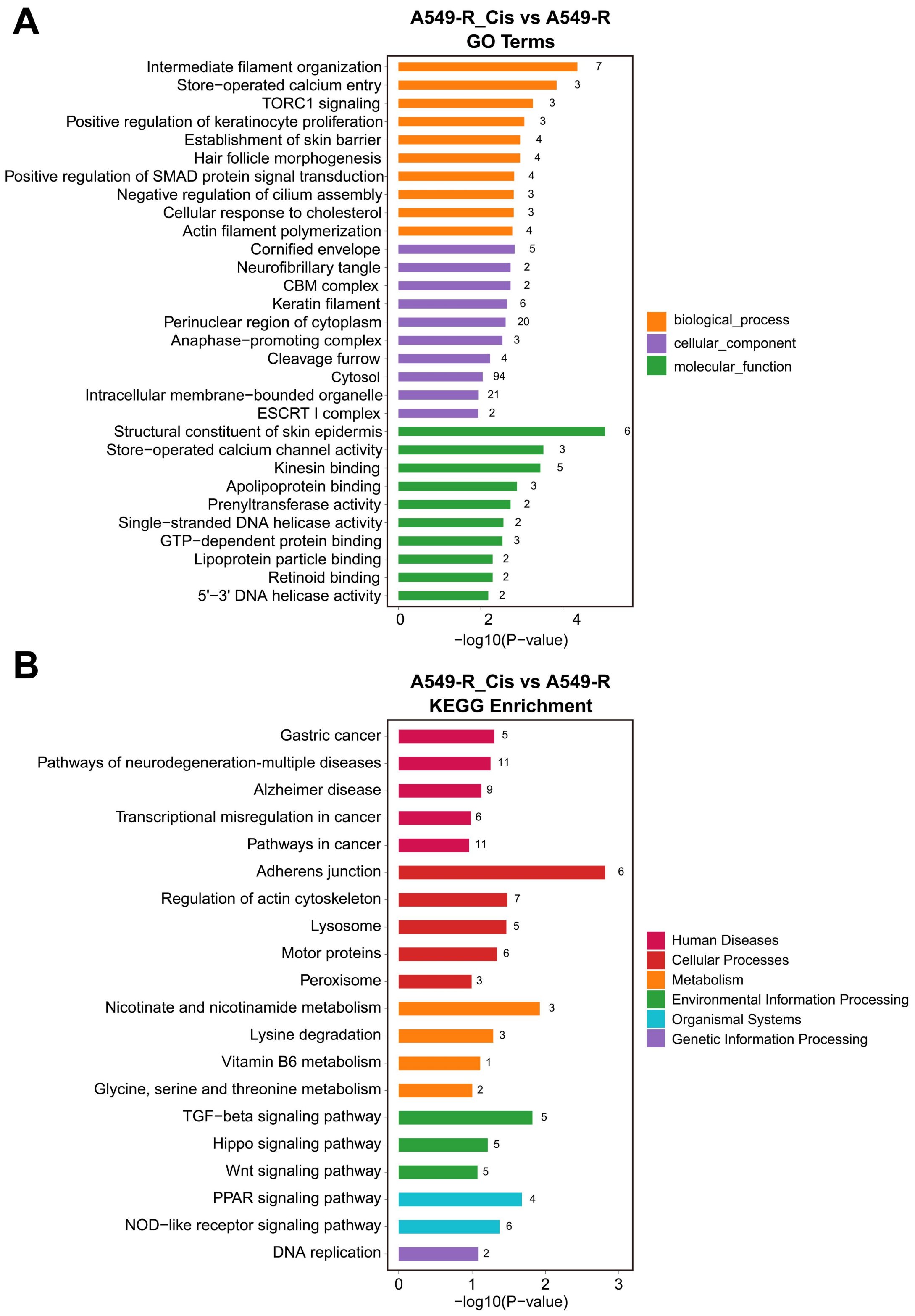

Figure S19. (A) GO and (B) KEGG enrichment analysis of differential expression proteins (DEPs).

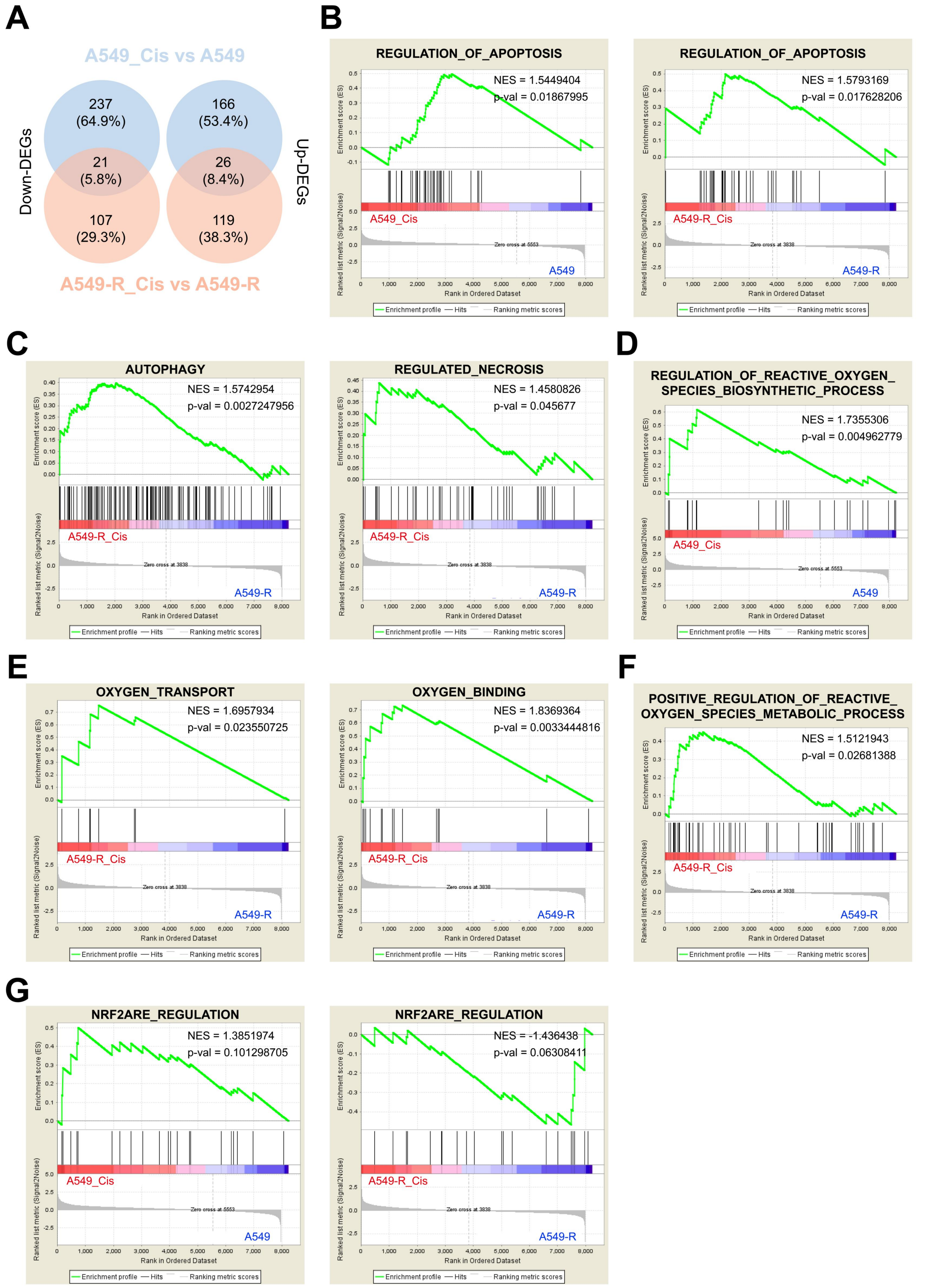

Figure S20. (A) The venny gram of common DEPs in A549-R_Cis vs A549-R and A549_Cis vs A549. (B) Cisplatin administration in A549 and A549-R cells was positively correlated with apoptosis. (C) In A549-R, cisplatin administration was positively correlated with autophagy and necrosis. (D) In A549, cisplatin administration was positively correlated with ROS biosynthesis

pathway. In A549-R, cisplatin administration was positively correlated with (E) the pathway of oxygen and (F) ROS metabolic pathway. (G) Cisplatin administration tended to activate Nrf2 pathway in A549 cells (no statistical significance), and tended to inhibit Nrf2 pathway in A549-R cells (no statistical significance).

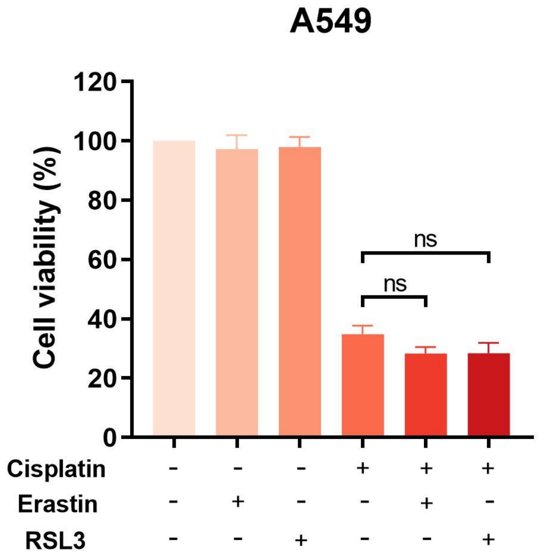

Figure S21. Survival rate of cisplatin with or without ferroptosis inducers (erastin, RSL3) in combination.

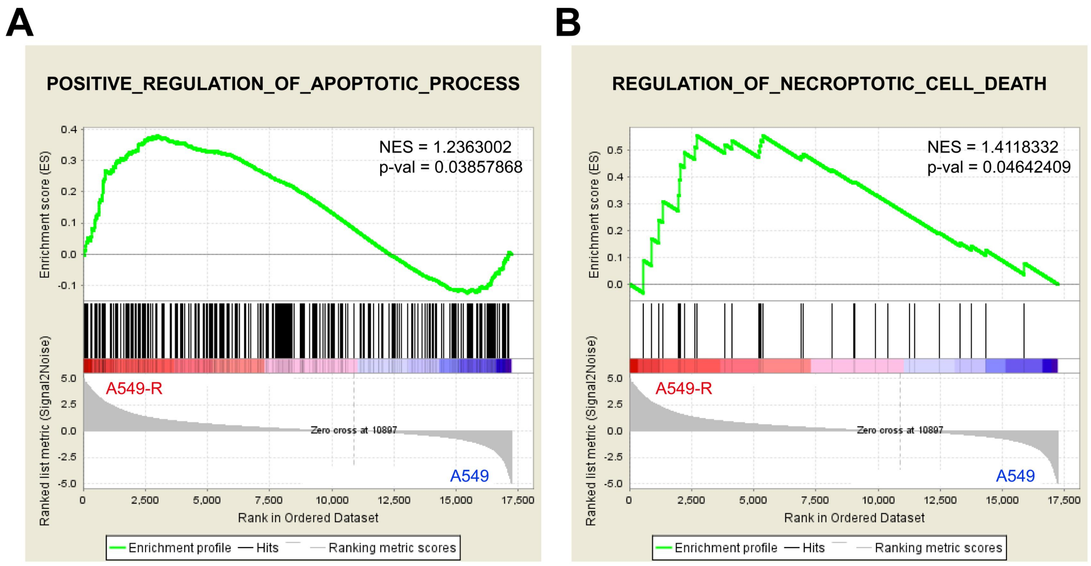

Figure S22. DEGs (A549-R vs A549) was positively correlated with (A) apoptosis and (B) necroptosis.

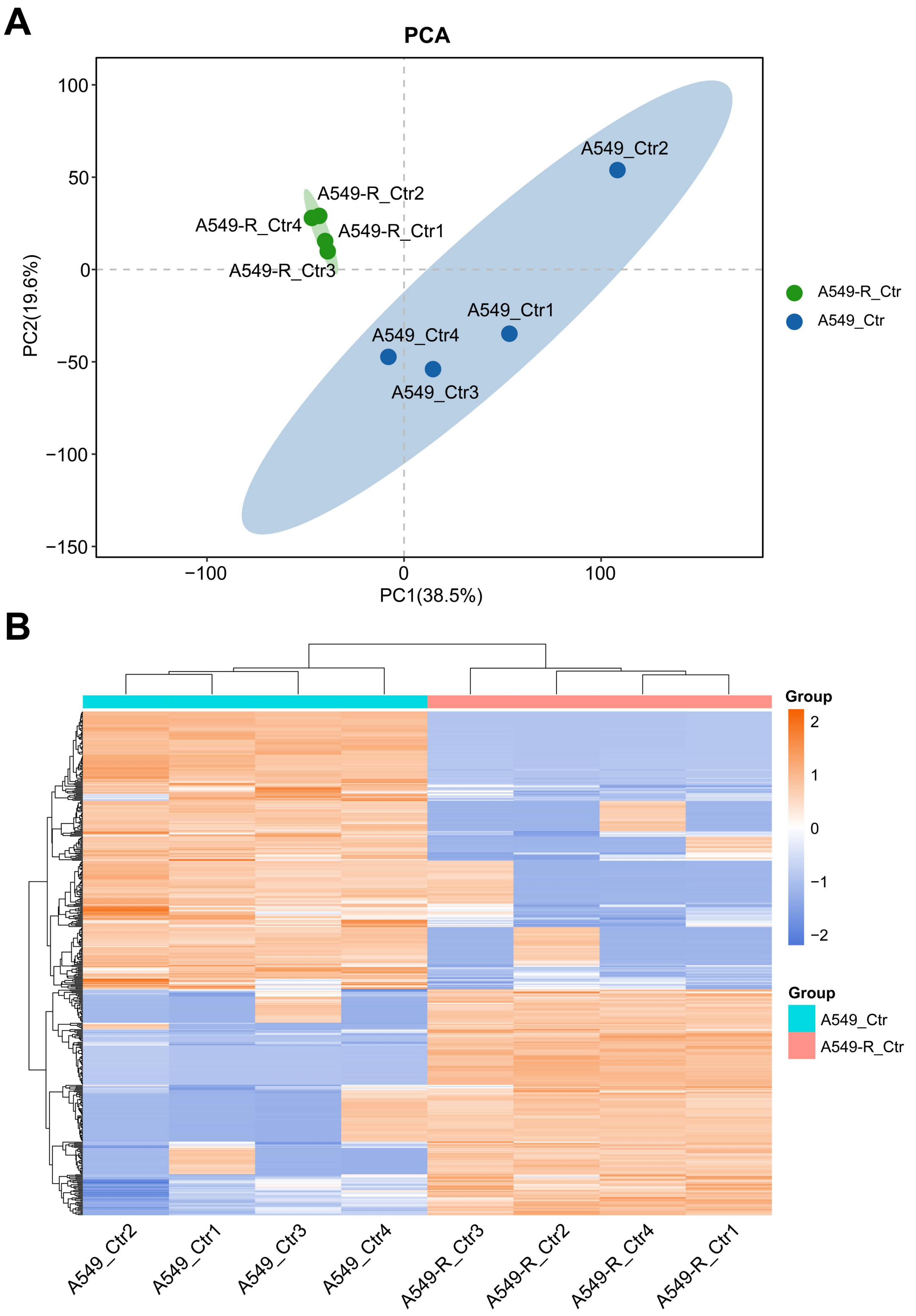

Figure S23. (A) PCA of transcriptome data of A549-R and A549 cell lines. (B) The heatmap of DEGs (A549-R vs. A549).

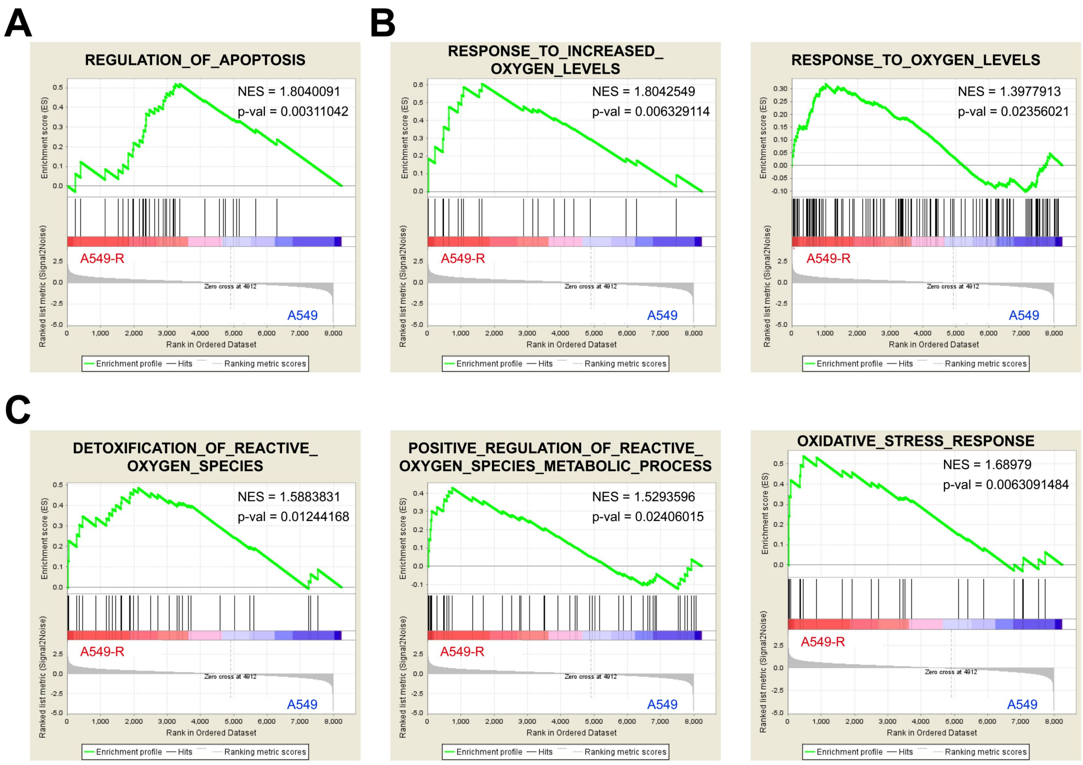

Figure S24. DEPs (A549-R vs. A549) was positively correlated with (A) apoptosis, (B) oxygen and (C) ROS.

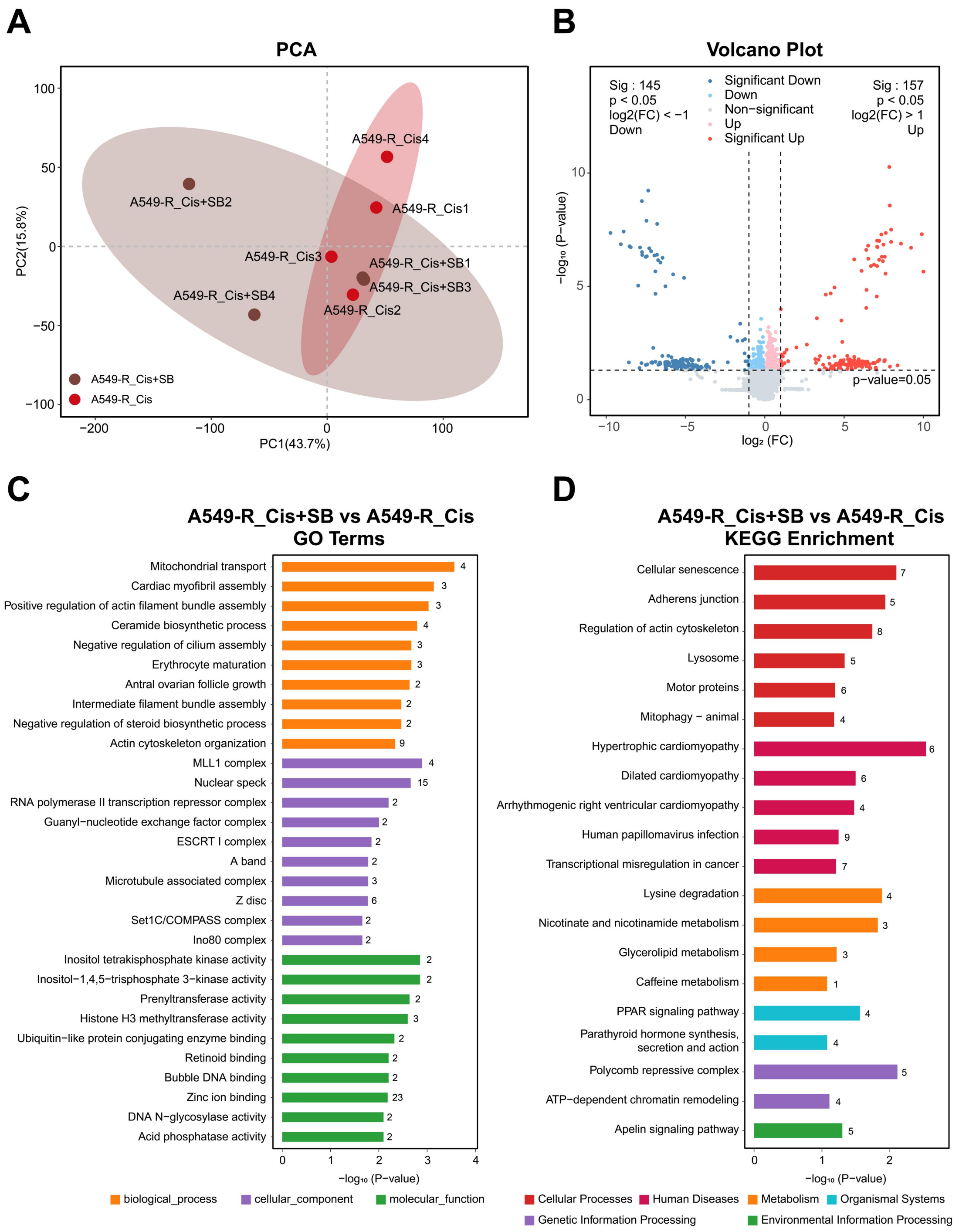

Figure S25. (A) PCA and (B) volcano plot of proteomic data of A549-R_Cis+SB 202190 and A549-R_Cis. (C) GO and (D) KEGG enrichment analysis of DEPs in A549-R_Cis+SB 202190 and A549-R_Cis.

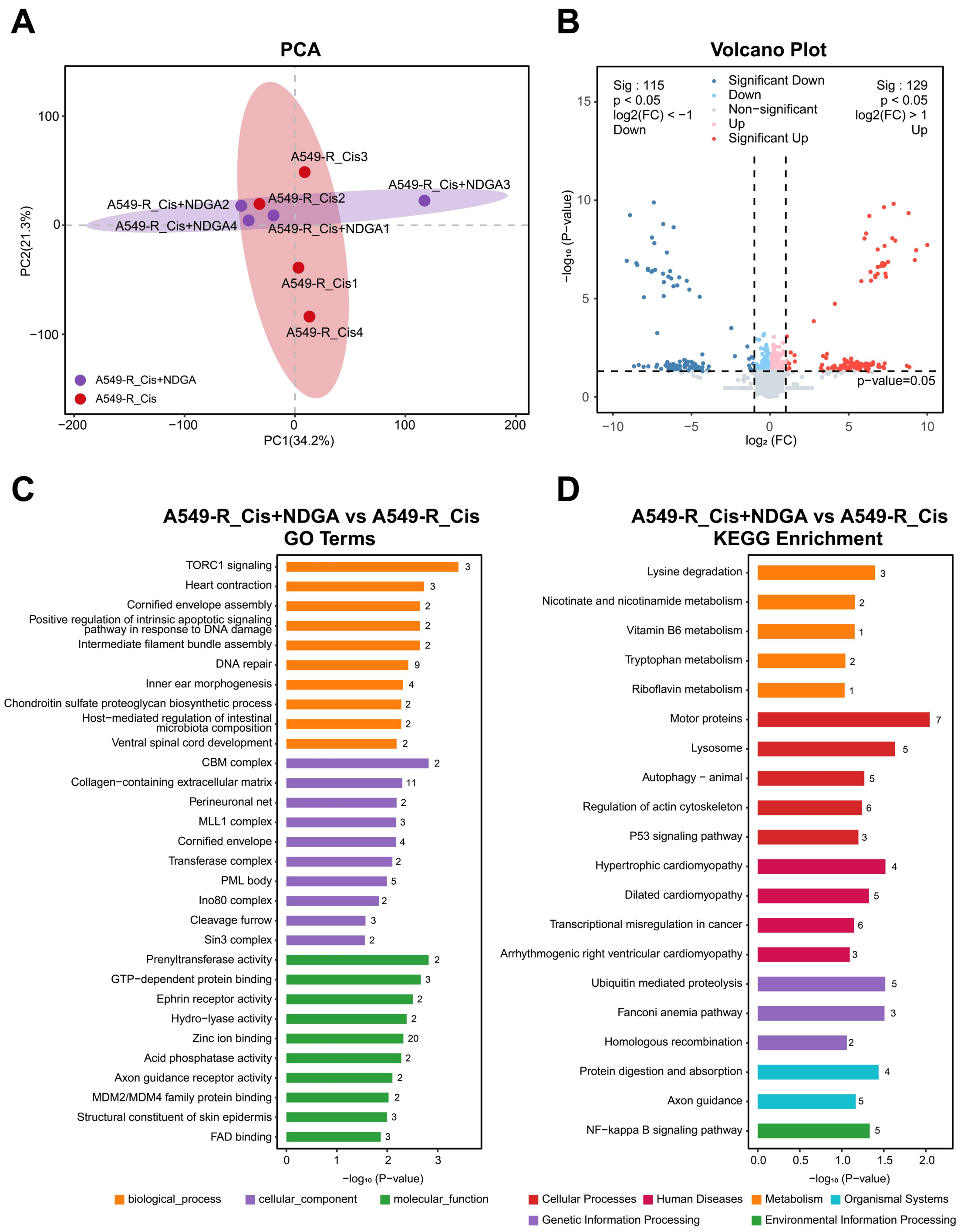

Figure S26. (A) PCA and (B) volcano plot of proteomic data of A549-R_Cis+NDGA and A549-R_Cis. (C) GO and (D) KEGG enrichment analysis of DEPs in A549-R_Cis+NDGA and A549-R_Cis.

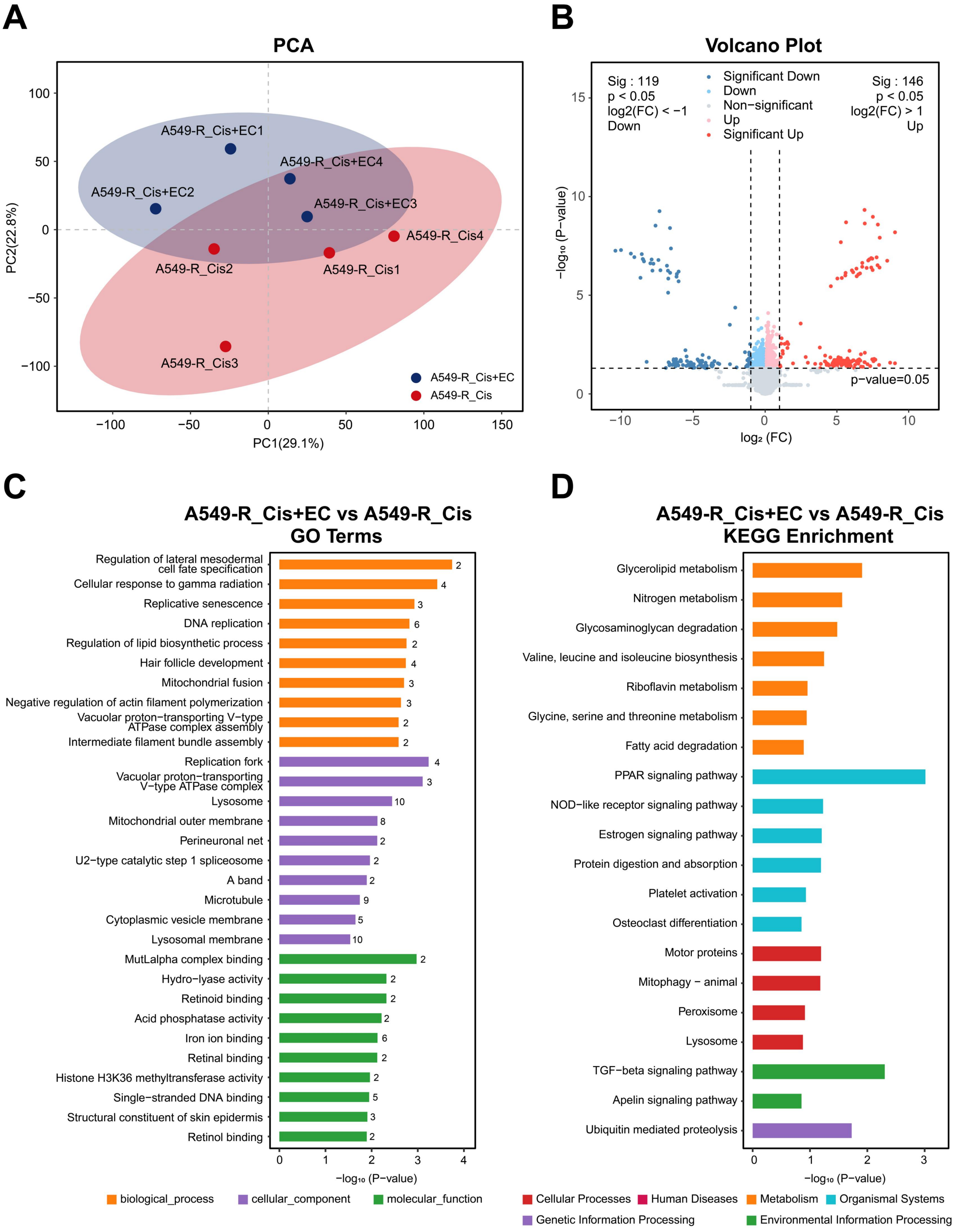

Figure S27. (A) PCA and (B) volcano plot of proteomic data of A549-R_Cis+EC and A549-R_Cis. (C) GO and (D) KEGG enrichment analysis of DEPs in A549-R_Cis+EC and A549-R_Cis.

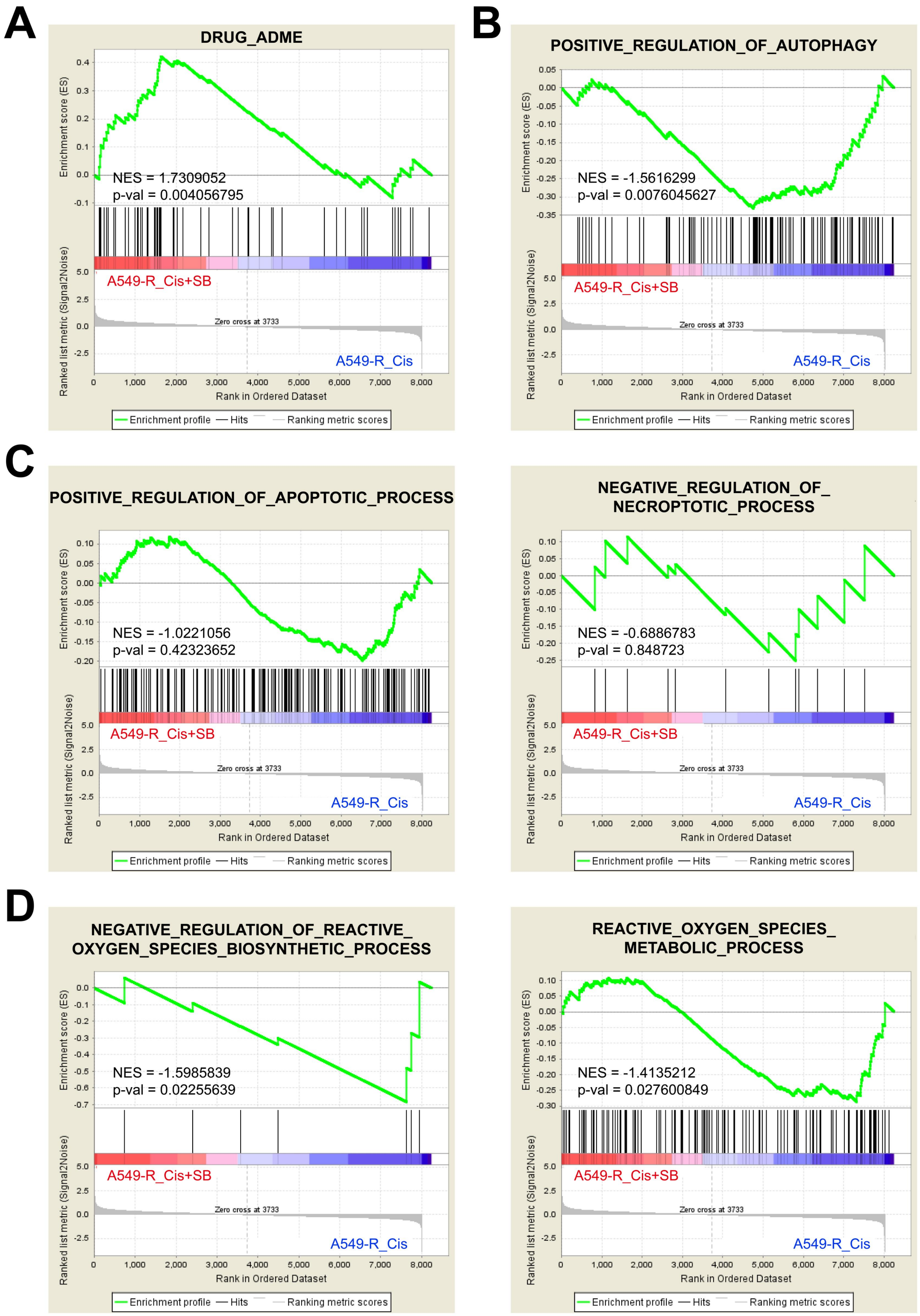

Figure S28. GSEA analysis of DEPs (A549-R_Cis+SB 202190 vs A549-R_Cis). Compared with cisplatin administration, cisplatin combined with SB 202190 group was associated with (A) drug ADME, (B) inhibited autophagy, (C) but did not affect apoptosis and necroptosis. Compared with cisplatin administration, cisplatin combined with SB 202190 up-regulated ROS biosynthetic

pathway and down-regulated ROS metabolic pathway.

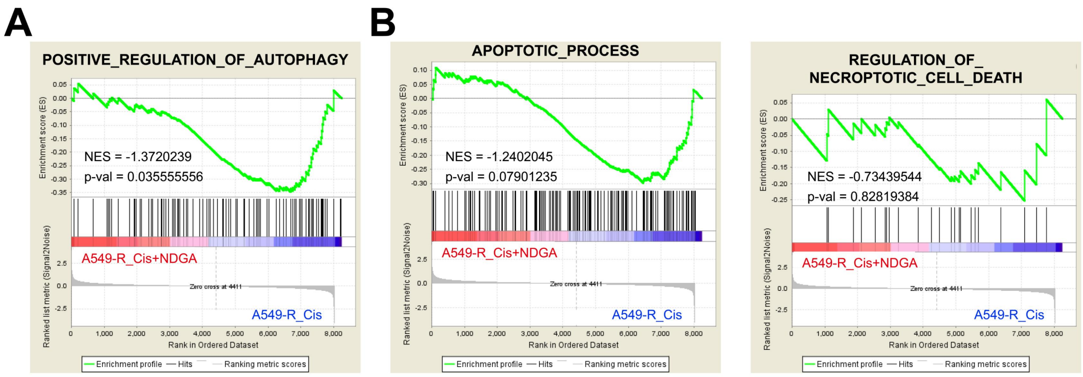

Figure S29. GSEA analysis of DEPs (A549-R_Cis+NDGA vs A549-R_Cis). Compared with cisplatin administration, cisplatin combined with NDGA (A) inhibit autophagy, (B) but did not affect apoptosis and necroptosis.

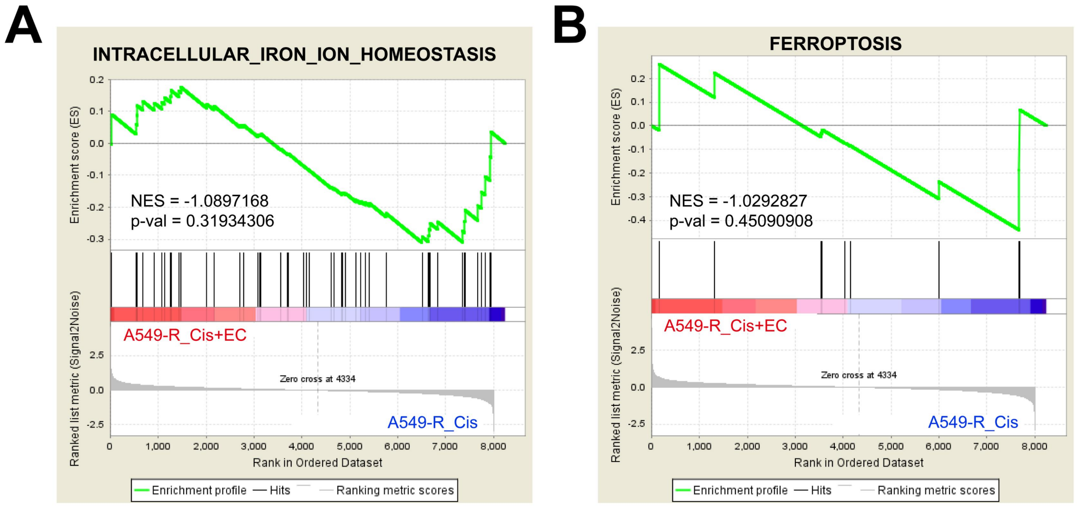

Figure S30. GSEA analysis of DEPs (A549-R_Cis+EC vs A549-R_Cis). Compared with cisplatin administration, cisplatin combined with EC had no significant correlation with iron ion homeostasis and ferroptosis.

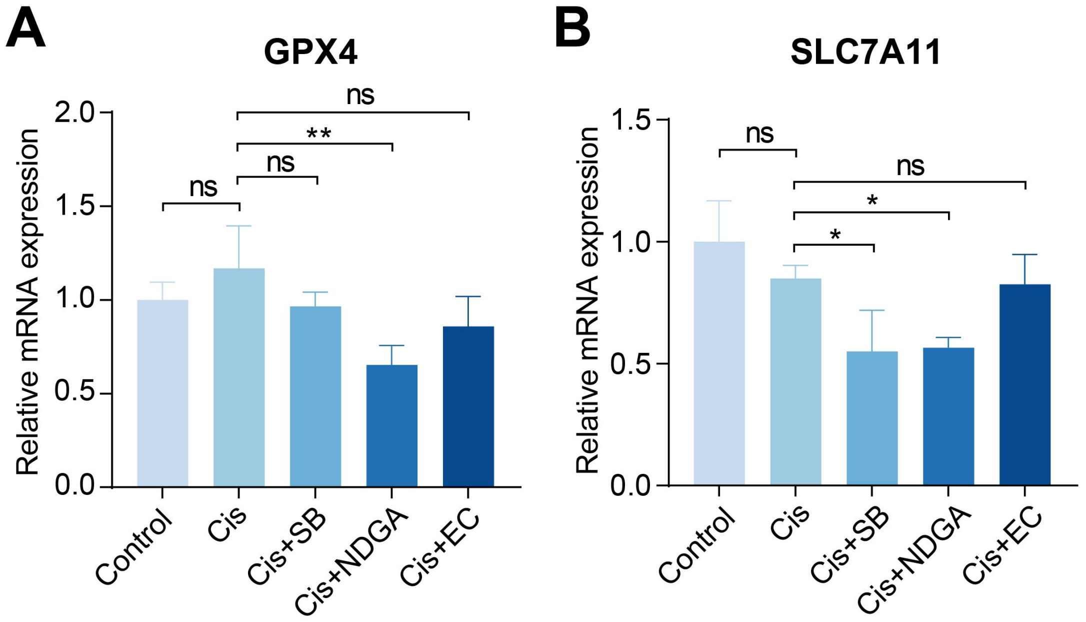

Figure S31. The relative mRNA expression of (A) GPX4 and (B) SLC7A11 after the administration of cisplatin with/without candidate compounds. All experiments were independently repeated at least three times.

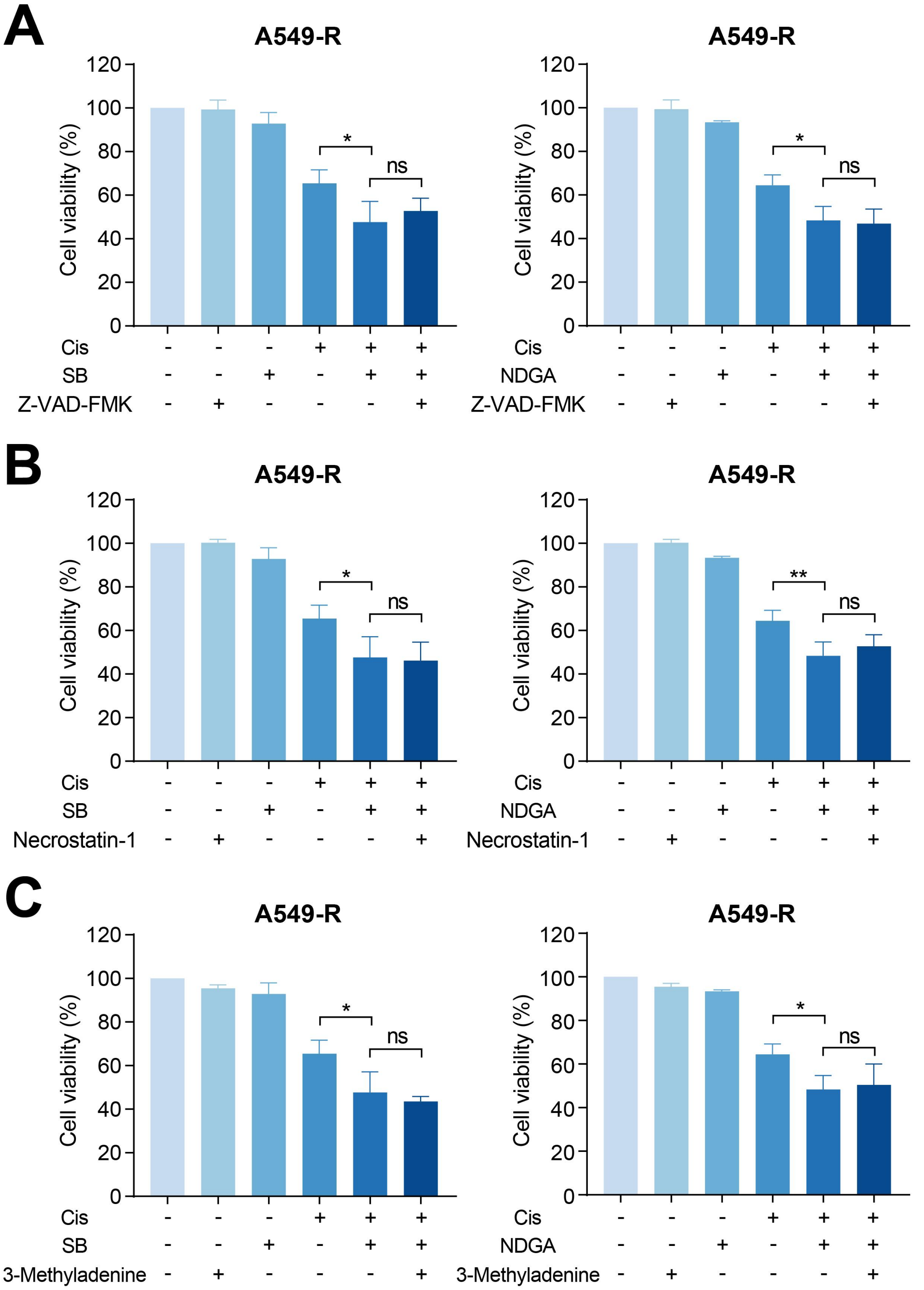

Figure S32. Effect of other death inhibitors on cell death induced by cisplatin combined with SB 202190 or NDGA. (A) Apoptosis inhibitors (Z-VAD-FMK), (B) necroptosis inhibitors (Necrostatin-1), or (C) autophagy inhibitors (3-Methyladenine) were used in combination with cisplatin/cisplatin+candidate compounds. All experiments were independently repeated at least

three times.

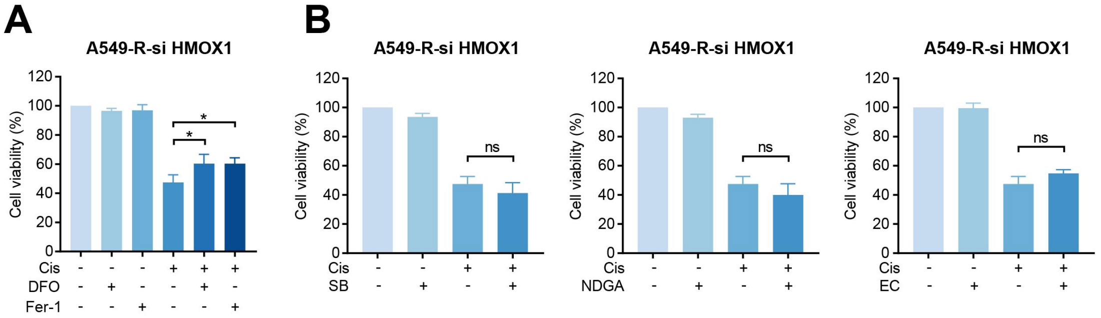

Figure S33. Effect of knocking down HMOX1 on ferroptosis inhibitors or candidate compounds. (A) Fer-1 and DFO inhibited the cell death induced by cisplatin after HMOX1 knockdown. (B) The combination of the candidate compounds with cisplatin did not increase the cell mortality rate compared to cisplatin monotherapy after HMOX1 knockdown. All experiments were independently repeated at least three times.

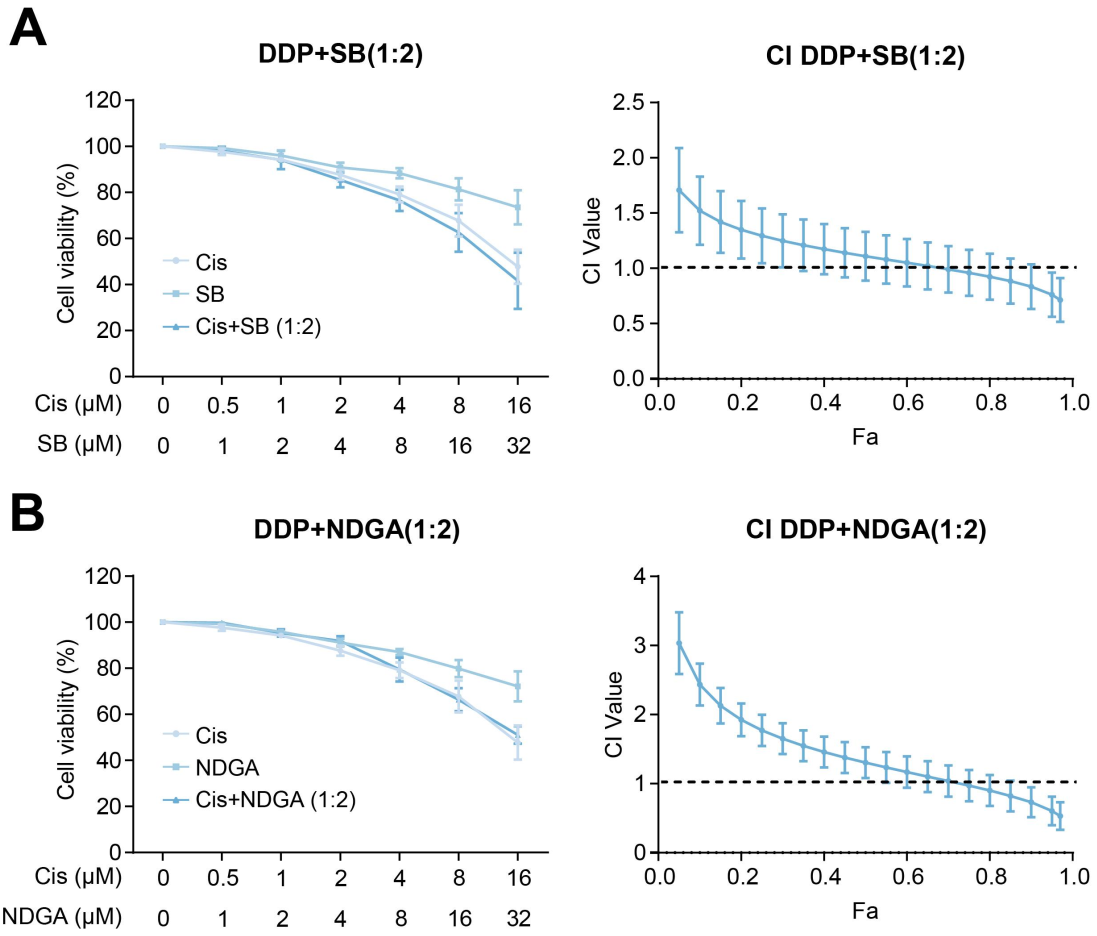

Figure S34. Effect and CI value of SB 202190 or NDGA combined with cisplatin after HMOX1

knockdown. All experiments were independently repeated at least three times.
